# Expression of the TPα and TPβ isoforms of the thromboxane prostanoid receptor (TP) in prostate cancer: clinical significance and diagnostic potential

**DOI:** 10.18632/oncotarget.12256

**Published:** 2016-09-26

**Authors:** Eamon P. Mulvaney, Christine Shilling, Sarah B. Eivers, Antoinette S. Perry, Anders Bjartell, Elaine W. Kay, R. William Watson, B. Therese Kinsella

**Affiliations:** ^1^ UCD School of Biomolecular and Biomedical Science, University College Dublin, Belfield, Dublin, Ireland; ^2^ UCD School of Medicine, UCD Conway Institute of Biomolecular and Biomedical Research, University College Dublin, Belfield, Dublin, Ireland; ^3^ Department of Pathology, Beaumont Hospital and Royal College of Surgeons, Dublin, Ireland; ^4^ Department of Translational Medicine, Division of Urological Cancers, Skåne University Hospital Malmö, Lund University, Sweden

**Keywords:** thromboxane, receptor, prostate, cancer, prostanoid

## Abstract

The prostanoid thromboxane (TX) A_2_ plays a central role in haemostasis and is increasingly implicated in cancer progression. TXA_2_ signals through two T Prostanoid receptor (TP) isoforms termed TPα and TPβ, with both encoded by the *TBXA2R* gene. Despite exhibiting several functional and regulatory differences, the role of the individual TP isoforms in neoplastic diseases is largely unknown.

This study evaluated expression of the TPα and TPβ isoforms in tumour microarrays of the benign prostate and different pathological (Gleason) grades of prostate cancer (PCa). Expression of TPβ was significantly increased in PCa relative to benign tissue and strongly correlated with increasing Gleason grade. Furthermore, higher TPβ expression was associated with increased risk of biochemical recurrence (BCR) and significantly shorter disease-free survival time in patients post-surgery. While TPα was more variably expressed than TPβ in PCa, increased/high TPα expression within the tumour also trended toward increased BCR and shorter disease-free survival time. Comparative genomic CpG DNA methylation analysis revealed substantial differences in the extent of methylation of the promoter regions of the *TBXA2R* that specifically regulate expression of TPα and TPβ, respectively, both in benign prostate and in clinically-derived tissue representative of precursor lesions and progressive stages of PCa. Collectively, TPα and TPβ expression is differentially regulated both in the benign and tumourigenic prostate, and coincides with clinical pathology and altered CpG methylation of the *TBXA2R* gene. Analysis of TPβ, or a combination of TPα/TPβ, expression levels may have significant clinical potential as a diagnostic biomarker and predictor of PCa disease recurrence.

## INTRODUCTION

As one of the most common cancers among men, prostate cancer (PCa) is a leading cause of death within this population across the developed world [1]. From the clinical standpoint, the vast majority of prostate tumours remain asymptomatic, and in most cases an active surveillance approach is followed [1, 2]. Treatments for what are deemed as the more significant or progressive cases include surgery, radiation therapy, *anti-* androgen therapies and chemotherapy [1]. However, following surgery or radiation therapy, approximately one third of patients with localised PCa experience disease relapse, typically detected by a rise in serum prostate-specific antigen (PSA) levels, termed “biochemical recurrence (BCR)” or PSA failure [3]. Furthermore, despite the initial success of androgen-deprivation therapies (ADTs), many patients eventually fail in this and the disease develops to the incurable metastatic castrate-resistant PCa (CRPC) stage, for which only palliative treatments are typically advised [4]. Hence, a key goal of current research is to understand the mechanistic basis of the disease and to identify biomarkers that will discriminate between the indolent conditions and the more aggressive or fatal forms of PCa, allowing clinicians and patients to make more informed decisions on the optimal treatment approaches [2].

By now numerous epidemiological studies have shown that regular intake of the non-steroidal *anti-*inflammatory drug (NSAID) aspirin, an inhibitor of the cyclooxygenases (COXs), substantially reduces risks in both the incidence and progression of several prevalent cancers, including PCa [5–7]. While those studies do not specify which COX-1/2 metabolite(s) are actually lowered by aspirin accounting for its prophylactic benefits, recent studies suggest that some/much of its *anti-*cancer effects may be due to its ability to inhibit thromboxane (TX) A_2_ generation, a prostanoid more typically implicated in thrombosis and cardiovascular disease. Increased levels of TXA_2_ and expression of its synthase and its T prostanoid receptor, the TP, have been found in a number of prevalent cancers [8] and, mechanistically, the role of TXA_2_ in cancer may be explained by the ability of the TXA_2_-TP axis to regulate key mitogenic/extracellular signal regulated kinase (ERK)- and RhoA-mediated signalling cascades that contribute to tumour development and metastasis [9, 10]. For example, increased levels of TXA_2_, TXA_2_ synthase and the TP strongly correlate with bladder [11], prostate [12, 13], colorectal [14, 15] and non-small cell lung cancer [16]. Furthermore, it has long been known that platelets, the main source of TXA_2_ and key target of aspirin, play a central role in tumour progression promoting cancer cell metastasis, immune evasion and extravasation [17].

In humans, TXA_2_ actually signals through two isoforms of the TP, termed TPα and TPβ, which have both overlapping but also distinct physiological roles, exhibiting several critical differences in their modes of signalling and regulation [10, 18–23]. While TPα and TPβ are encoded by the same *TBXA2R* gene, they are differentially expressed in a range of tissues due to their transcriptional regulation by different Promoters, designated Prm1 and Prm3, respectively, within the *TBXA2R* [24–27]. Functionally, both TPα and TPβ couple to Gα_q_-mediated phospholipase (PL) Cβ activation, their primary effector, but also readily couple to activation of the ERK and Gα_12_-RhoA-signalling cascades promoting cell proliferation, mitogenesis, and the dynamic changes that drive cell migration and metastasis [18, 28]. In more recent studies, it was also discovered that both TPα and TPβ directly interact with and regulate signalling by protein kinase C-related kinase/protein kinase novel (PRK/PKN) [19], a family of 3 AGC kinases and RhoA effectors that act immediately downstream of phosphatidyl inositol (Pi) 3′kinases and strongly, yet differentially, implicated in several cancers [29–31]. Indeed, in the specific context of PCa, activation of the PRKs (e.g. PRK1) in response to androgen receptor (AR) signalling catalyses phosphorylation of histone (H)3 at Thr11 (H3pThr11) which, in-turn, serves as a specific epigenetic marker and *gatekeeper of androgen induced-chromatin remodelling and -gene expression within the prostate* [19, 32–34]. Furthermore, similar to the AR, TP-mediated PRK1 activation not only leads to H3Thr11 phosphorylation in response to TXA_2_ but can also cooperate with the AR to enhance androgen induced -chromatin remodelling (H3pThr11) and -transcriptional activation/gene expression within the prostate [19]. Hence, similar to the androgens, these studies suggested that TXA_2_, through its ability to directly regulate PRK-induced H3pThr11, is a strong epigenetic regulator thereby adding to the range of possible mechanisms whereby the aspirin-target TXA_2_ may influence neoplastic growth. Added to this complexity, the TPα and TPβ isoforms appear to differentially associate with- and regulate-signalling by the individual PRKs (PRK1/PKNα, PRK2/PKNγ, PRK3/PKNβ), suggesting clear functional differences between TPα and TPβ within the prostate and, potentially, in PCa [35].

As stated, while a number of studies have reported linkages between increased TXA_2_ signalling and TP expression with certain neoplasms [9–15], to date such studies have not investigated the role of the individual TPα and TPβ isoforms in those diseases. Hence, in view of the clear functional and regulatory differences between TPα and TPβ, coupled with the discovery of their ability to regulate the PRK-signalling cascade implicated in PCa etiology, and in a TP isoform-specific manner, the aim of this study was to histologically evaluate expression of the individual TPα and TPβ isoforms in clinical prostatectomy specimens representative of the benign prostate and of different pathological (Gleason) grades of PCa. Furthermore, it was aimed to investigate whether TPα and/or TPβ expression might serve as surrogate biomarker(s) in PCa, correlating with Gleason grade, pathologic tumour staging (PTS) or with significant clinical outcomes, such as patient progression to BCR, potential for disease relapse and/or the development of the more severe aggressive forms of PCa.

## RESULTS

### Expression of TPα and TPβ in benign prostate and prostate tumour tissue

Expression of the individual TPα and TPβ isoforms was initially evaluated through IHC analysis in a series of full-face radical prostatectomy specimens (*N* = 17) using affinity-purified *anti-*TPα or *anti-*TPβ isoform-specific antibodies [10, 36]. Validation of antibody specificity was confirmed whereby pre-incubation of the *anti-*TPα antiserum with the cognate TPα, but not with the TPβ, antigenic peptide blocked all *anti-*TPα IHC staining while pre-incubation of the *anti-*TPβ antiserum with the cognate TPβ, but not with the TPα, antigenic peptide competed all *anti-*TPβ staining (Supplementary Figure S1A). Validation of the TP isoform-specific antibodies was also demonstrated whereby the individual *anti*-TPα and *anti*-TPβ antibodies specifically and exclusively immunoprecipitated only their cognate receptor isoform from HEK 293 cell lines expressing TPα or TPβ (Supplementary Figure S1B).

**Figure 1:**
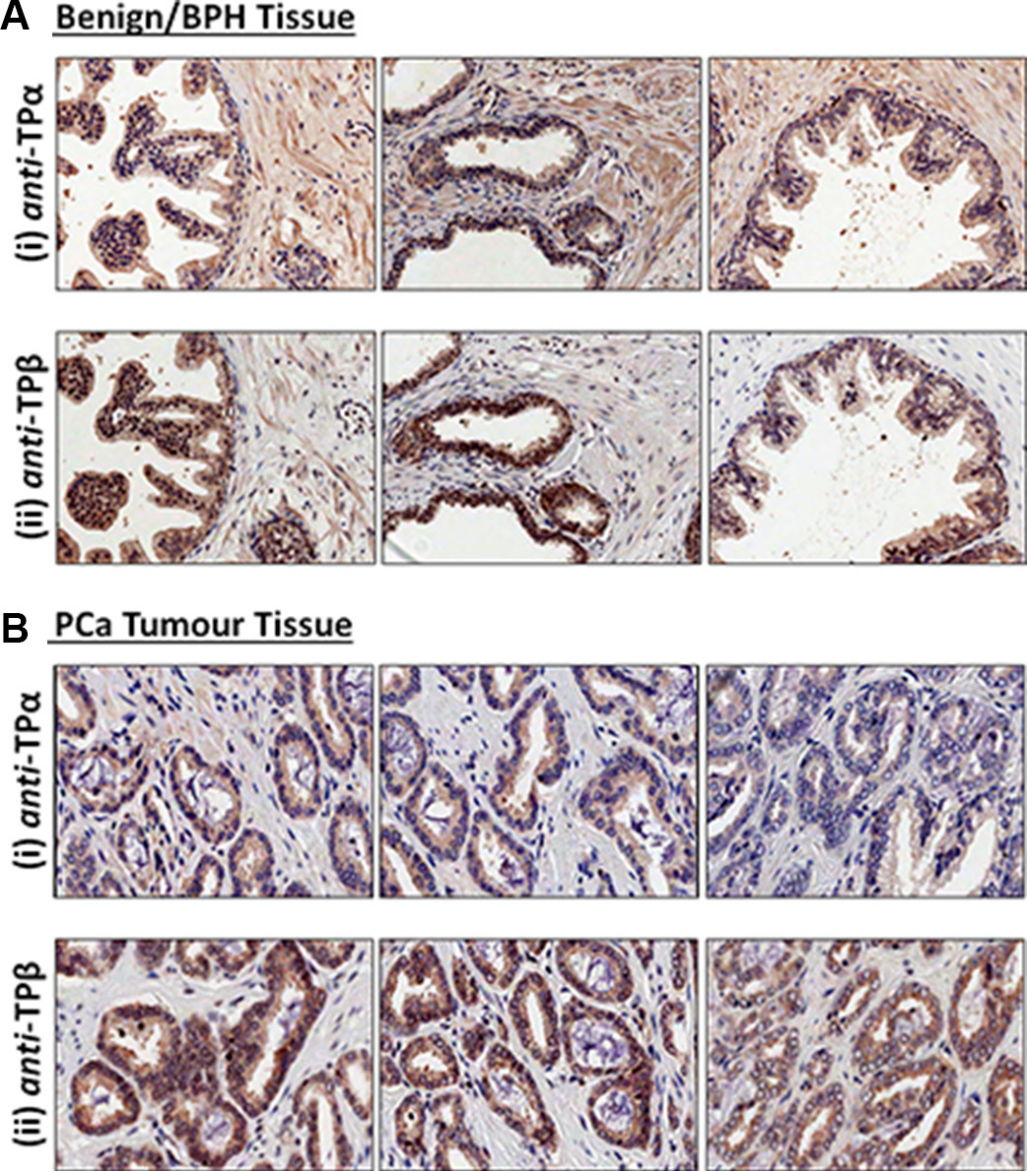
Expression of TPα and TPβ in benign prostate and PCα tissue Representative IHC analysis of TPα and TPβ protein expression in serial sections of paraffin-embedded, formalin-fixed full-face prostate tissues screened with affinity-purified *anti-*TPα or *anti-*TPβ antibodies. In all, 17 full-face sections were examined. Expression of TPα and TPβ was evident in the prostate glandular luminal epithelial cells and in the fibromuscular stromal smooth muscle cells where expression of both TPα and TPβ was predominantly cytoplasmic. In certain cases, nuclear expression of TPβ was also observed in the luminal epithelial cells. *Panel* (**A**) Benign prostate. *Panel* (**B**) Prostate tumour. All sections were counterstained with haematoxylin and images shown were captured at 200× magnification.

In all prostatectomy specimens, both TPα and TPβ were expressed in the fibromuscular stroma, consistent with the contractile role of TXA_2_ in the prostate, and also in the luminal epithelial cells lining the secretory ducts (Figure 1A and Supplementary Figure S1C). In the benign tissue, expression of TPα was more predominant than TPβ in the stromal or smooth muscle regions (Figure 1A(i)) while, conversely, expression of TPβ was typically stronger than that of TPα within the glandular epithelium (Figure 1A(ii)). While expression of both TPα and TPβ was predominantly cytoplasmic (Figure 1A), nuclear staining of TPβ, and to a much lesser extent of TPα, was also observed within the glandular epithelial cells in certain specimens (Figure 1A and Supplementary Figure S1C). Within the tumour epithelium, TPβ was again more strongly expressed than TPα, and there was a tendency toward increased expression of TPβ, and to a lesser extent TPα, in the tumour relative to the benign epithelium in sections from the same patient (Figure 1B and Supplementary Figure S1C). In the stromal regions of the prostate tissue, no substantial differences in either the cytoplasmic or nuclear expression of TPα or TPβ were evident between the benign and tumour regions.

### Correlation of TPα and TPβ expression with gleason grading

The Gleason grade is the most common pathologic grading system for PCa, and remains one of the most useful prognostic tools in assessing disease severity, risk of clinical recurrence, and often informs the specific treatment regime to be followed [37]. Bearing in mind the mounting evidence for the role of the TXA_2_ –TP axis in cancer progression but also of the clear functional and regulatory differences between the TPα and TPβ isoforms [10, 18–23], it was deemed imperative to investigate expression of the individual TPα and TPβ isoforms in PCa and to establish whether cytoplasmic expression of either isoform within the glandular epithelium might correlate with Gleason grade.

**Figure 2:**
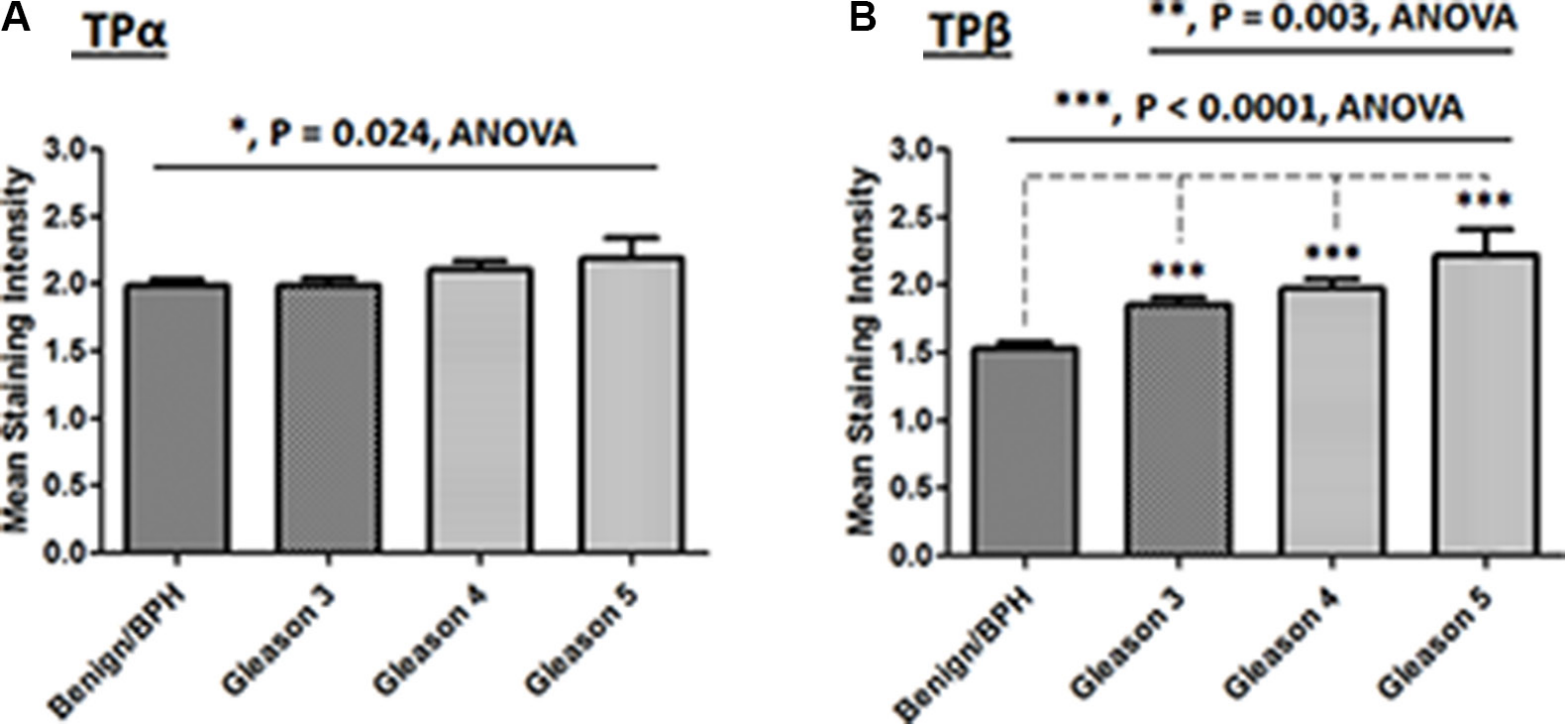
Correlation of TPα and TPβ staining intensity with Gleason grading in the PCRC TMA Comparison of TPα (*Panel* **A**) and TPβ (*Panel* **B**) immunohistochemical staining intensities in the “PCRC TMA”, where data is presented as the mean TPα or TPβ score (Range, 0–3) for each particular histology present (± SEM). Differences were analysed by one-way ANOVA group comparisons (solid black line) with individual *post-hoc* Bonferroni's Multiple Comparison Tests (dotted line) and the asterisks indicate where scoring of TPα (*Panel A*) or TPβ (*Panel B*) staining intensity within the PCa tissue is significantly altered between the groups indicated, or compared to the Benign/BPH tissue, where *, ** and *** indicate *P* < 0.05, 0.01 and 0.001, respectively.

Initially expression of TPα and TPβ was evaluated using IHC in the “PCRC TMA” patient cohort and, in each case, mean expression levels of both TPα and TPβ differed substantially in the PCa relative to the benign tissue (Figure 2). Specifically, in the case of TPα, alteration in its expression levels between the benign/BPH versus Gleason grade was variable, whereby some patients showed either no change, while others showed an increase and others an actual decrease in TPα expression in benign versus PCa epithelium. Hence, due to this variability, the only notable quantifiable difference in TPα expression across the “PCRC TMA” was a modest but significant increase between the benign versus all Gleason groupings (Benign/BPH versus Gleason 3/4/5; *P* = 0.024; Figure 2A). Furthermore, while two-way contingency analyses (Fisher exact) showed that there was no significant difference in TPα expression between benign/BPH and a combined Gleason 3/4/5 cohort, there was a marginal, but significant *nett* decrease noted between benign/BPH and Gleason 3 groupings (*P* = 0.0458; Table 1A). In the case of TPβ, its expression in the epithelium was significantly increased between benign/BPH versus Gleason 3, Gleason 4, Gleason 5 (*P* < 0.001, all; Figure 2B). In addition, there was a significant increase in TPβ expression between the Gleason 3 versus Gleason 5 groupings (*P* = 0.003; Figure 2B). Furthermore, contingency analysis showed that there was a highly significant increase in TPβ expression between benign/BPH versus Gleason 3/4/5, Gleason 3 alone, and also Gleason 4/5 (*P* < 0.0001, all; Table 1B). There was also a significant increase in TPβ expression between the benign/BPH/Gleason 3 versus Gleason 4/5 groupings (*P* < 0.0001; Table 1B). While nuclear staining of TPβ, and to a much lesser extent of TPα, was also observed within the glandular epithelium in certain TMA cores, this was not a consistent feature and did not correlate with Gleason grade in either case (Data not shown).

**Table 1A:** “PCRC TMA” - TPa staining intensity/gleason grading correlation

Contingency Test	Histology	Low Expression	High Expression	*P* Value^$^
		No. (%)	No. (%)	
**Histologically benign versus tumour (G3, G4 or G5)**	Benign/BPH (*n*^#^ = 378)	79 (21)	299 (79)	*P* = 0.1870
	Gleason 3 + 4 + 5 (*n*= 604)	149 (25)	455 (75)	
**Histologically benign versus Gleason grade 3**	Benign/BPH (*n* = 378)	79 (21)	299 (79)	*P = 0.0458
	Gleason 3 (*n* = 299)	83 (28)	216 (72)	
**Histologically benign versus high grade tumour (G4 or G5)**	Benign/BPH (*n* = 378)	79 (21)	299 (79)	*P* = 0.8509
	Gleason 4 + 5 (*n* = 305)	66 (22)	239 (78)	
**Benign & Gleason 3 versus high grade tumour (G4 or G5)**	Benign/BPH+Gleason 3 (*n* = 677)	162 (24)	515 (76)	*P* = 0.4628
	Gleason 4 + 5 (*n* = 305)	66 (22)	239 (78)	

**Table 1B:** “PCRC TMA” - TPβ staining intensity/gleason grading correlation

Contingency Test	Histology	Low Expression	High Expression	*P* Value^$^
		No. (%)	No. (%)	
**Histologically benign versus tumour (G3, G4 or G5)**	Benign/BPH (*n*^#^ = 364)	184 (51)	180 (49)	***P < 0.0001
	Gleason 3 + 4 + 5 (*n*= 602)	193 (32)	409 (68)	
**Histologically benign versus Gleason grade 3**	Benign/BPH (*n* = 364)	184 (51)	180 (49)	***P < 0.0001
	Gleason 3 (*n* = 301)	104 (35)	197 (65)	
**Histologically benign versus high grade tumour (G4 or G5)**	Benign/BPH (*n* = 364)	184 (51)	180 (49)	***P < 0.0001
	Gleason 4 + 5 (*n* = 301)	89 (30)	212 (70)	
**Benign & Gleason 3 versus high grade tumour (G4 or G5)**	Benign/BPH+Gleason 3 (*n* = 665)	288 (43)	377 (57)	***P < 0.0001
	Gleason 4 + 5 (*n* = 301)	89 (30)	212 (70)	

Footnotes:

# *n* refers to the number of cores in each grouping.

$ Fisher's Exact Test where

* and *** refer to *P* < 0.05 and 0.0001, respectively.

### Correlation of TPα and TPβ expression with disease-free survival

While data from the “PCRC TMA” showed differential associations between TPα and TPβ expression and pathological PCa Gleason grading, it was of interest to determine if there is a correlation between TPα/TPβ expression and patient outcome following surgery and, hence, establish whether TPα or TPβ expression might have a prognostic or even therapeutic value in the clinical setting. Hence, expression of TPα and TPβ was further validated in the “Malmö TMA”, a large patient cohort TMA for which 10-yr clinical follow-up history and pathological staging (PTS) data was also available. As previous, TPα and TPβ expression in the “Malmö TMA” was initially evaluated for correlation between benign/BPH versus tumour tissue (Table 2A). Consistent with findings from the “PCRC TMA”, contingency analysis showed that expression of TPβ in the “Malmö TMA” was significantly increased in the PCa relative to the benign tissue (*P* < 0.0001; Table 2A). However, expression levels of TPα were more variable and in fact, across the “Malmö TMA”, there was a *nett* decrease rather than increase in TPα expression in PCa tissue relative to the benign/BPH tissue (*P* < 0.0001; Table 2A). Notably, these changes in TPα and TPβ expression also both correlated with Gleason score, wherein TPα staining intensity was found to be decreased, and that of TPβ to be increased, across increasing Gleason score within the patient cohort relative to the benign tissue (Supplementary Figure S2A and S2B and Supplementary Table S1A and S1B). Changes in TPα and TPβ expression within this cohort were also correlated with pathological staging, wherein TPα staining intensity was found to be unchanged but that of TPβ to be significantly increased between the organ-confined pT2 and the invasive extraprostatic pT3 staging (Supplementary Figure 2C and 2D and Supplementary Table S1C).

**Table 2A:** “Malmö TMA” - TP staining intensity/histology correlation

Contingency Test	Histology	Low Expression	High Expression	*P* Value^$^
		No. (%)	No. (%)	
TPα: Benign versus Tumour	Benign/BPH (*n*^#^ = 325)	88 (27)	237 (73)	***P < 0.0001
	Tumour (*n* = 252)	123 (49)	129 (51)	
TPβ: Benign versus Tumour	Benign/BPH (*n* = 323)	182 (56)	141 (44)	***P < 0.0001
	Tumour (*n* = 263)	99 (38)	164 (62)	

**Table 2B:** “Malmö TMA” - TP staining intensity/biochemical recurrence correlation

Contingency Test	Histology	Low Expression	High Expression	*P* Value^$^
		No. (%)	No. (%)	
TPα: Tumour Tissue	BCR Negative (*n*^&^ = 148)	78 (53)	70 (47)	*P* = 0.0570
BCR Negative vs BCR Positive	BCR Positive (*n* = 68)	26 (38)	42 (62)	
TPβ: Tumour Tissue	BCR Negative (*n*= 150)	62 (41)	88 (59)	**P = 0.0045
BCR Negative vs BCR Positive	BCR Positive (*n* = 73)	16 (22)	57 (78)	

**Table 2C:** “Malmö TMA” - TPα/TPβ staining intensity correlation in the tumour

Contingency Test	Histology	TPβ	TPβ	*P* Value^$^
		Low Expression	High Expression	
		No. (%)	No. (%)	
TPα vs TPβ Staining Intensity	TPα Low Expression (*n*^†^ = 122)	61 (50)	61 (50)	***P < 0.0001
	TPα High Expression (*n* = 129)	32 (25)	97 (75)	

Footnotes:

# *n* refers to the number of valid graded scores in the benign and tumour regions for patient cases.

& *n* refers to the number of patient cases in each grouping where biochemical recurrence (BCR) status is known.

† *n* refers to the number of patient cases in each grouping where valid TPα and TPβ scoring are both available.

$ Fisher's Exact Test where

** and *** refer to *P* < 0.01 and 0.0001.

At the time of reporting this study, approximately 30% of the Malmö patient cohort had progressed to BCR, defined as having a repeated serum PSA measurement of > 0.2 ng/ml. To determine whether TPα and/or TPβ expression levels may be a predictor of patient progression to BCR, the correlation of the BCR status (Positive/Negative) with low/high levels of TPα and TPβ expression within the tumour cores was next analysed (Table 2B). High expression of TPβ within the tumour cores correlated significantly with a positive BCR status (*P* = 0.0045; Table 2B). However, for TPα, as stated while its expression was more variable across the TMA, in those patient cases where its expression was actually increased in the tumour relative to the benign/BPH tissue there was a trend, albeit not significantly, toward a positive correlation between high TPα expression in tumour cores of patients that were BCR positive (*P* = 0.057; Table 2B).

**Figure 3:**
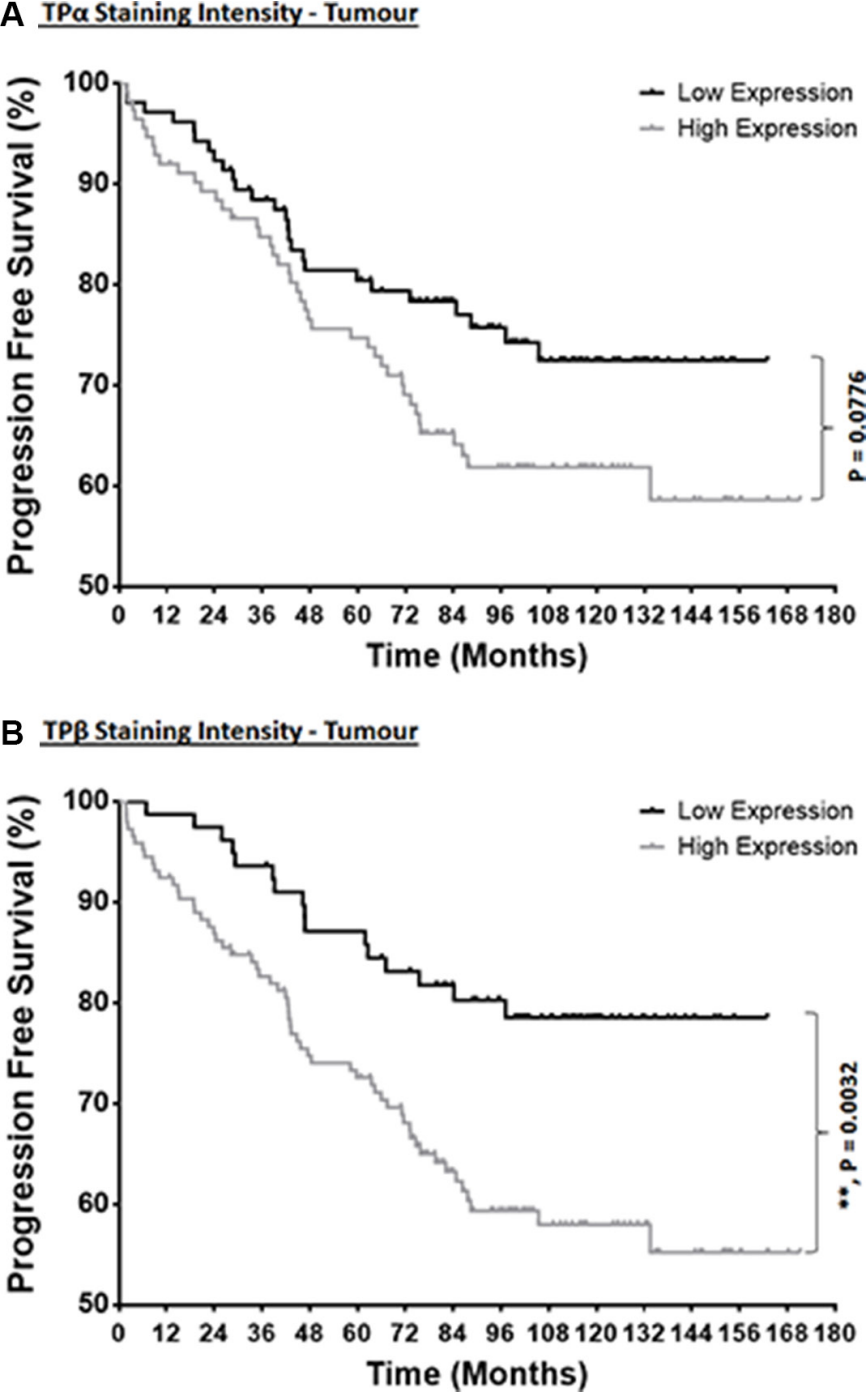
Correlation of TPα and TPβ expression with the progression to BCR Kaplan–Meier survival analysis of the correlation between low or high levels of TPα (*Panel* **A**) and TPβ (*Panel* **B**) expression with time to onset of BCR using the “Malmö TMA” dataset. BCR-free survival was compared between the expression groups by Kaplan–Mantel–Cox log rank comparison with Gehan–Breslow–Wilcoxon correction test, where ** indicates *P* < 0.01.

To determine whether TPα or TPβ expression level(s) is a significant predictor of BCR-free survival time following on from surgery, Kaplan–Meier survival curves were used to examine the relationship between low or high TPα or TPβ expression levels and disease-free/BCR-free time using the “Malmö TMA” (Figure 3). Through this Kaplan–Meier analyses, for TPα, while not reaching statistical significance (*P* = 0.0776), a trend was observed wherein patient cases showing high TPα expression were associated with a faster progression to BCR, in particular at 48–72 months' post-surgery (Figure 3A). Consistent with this trend, use of an ordinal scoring approach, as opposed to the binary low/high scoring approach, showed that increasing expression of TPα significantly associates with a faster progression to BCR (*P* = 0.0291; Supplementary Figure S3A). For TPβ, its expression strongly correlated with time-to-progression to BCR, wherein patients with high TPβ expression in the tumour had significantly shorter BCR-free time after surgery compared to patients with low TPβ expression (Figure 3B; *P* = 0.0032). This finding was noticeable from very early stages post-surgery and the survival divergence worsened significantly from approximately 40 months' post-surgery (Figure 3B). These findings for TPβ were also observed using the ordinal scoring approach (*P* = 0.0308; Supplementary Figure S3B).

To investigate whether expression levels of TPα and/or TPβ may add independent prognostic value over (i) Gleason scoring (GS) and (ii) pathological tumour staging (PTS) in the prediction of BCR-free survival time following surgery, additional multivariate time-to-event analyses were carried out (Supplementary Figure S4 and Supplementary Figure S5). As expected, GS strongly correlated with time-to-progression to BCR, wherein cases with increasing GS had progressively shorter BCR-free time (Supplementary Figure S4A; *P* < 0.0001). For TPα, multivariate analyses demonstrated a trend wherein increased or high TPα expression is associated with a shorter BCR-free time within certain GS groupings, in particular GS 5 cases (Supplementary Figure S4B; GS 5, *P* = 0.0272, *n* = 21; GS 6, *P* = 0.2003, *n* = 74; GS 7, *P* = 0.7201, *n* = 101; GS 9, *P* = 0.6254, *n* = 13). For TPβ, multivariate analyses demonstrated that its expression is also associated with a shorter BCR-free time within certain GS groupings, in particular GS 7 cases (Supplementary Figure 4C; GS 5, *P* = 0.0736, *n* = 20; GS 6, *P* = 0.6753, *n* = 75; GS 7, *P* = 0.0398, *n* = 106; GS 9, *P* = 0.3865, *n* = 15). In these analyses, the GS 8 and GS 10 datasets were excluded due to low numbers, precluding statistical analysis (GS 8, *n* = 3; GS 10, *n* = 1 for both TPα and TPβ). However, it is noteworthy that all these GS 8 and GS 10 cases showed high TPα and high TPβ expression and all rapidly progressed to BCR within five years. In the case of the second multivariate analysis (ii), as expected PTS strongly correlated with time-to-progression to BCR wherein cases with invasive pT3 staging had a significantly shorter BCR-free time compared to cases with organ-confined pT2 staging (Supplementary Figure S5A; *P* < 0.0001). In the case of TPα, this multivariate analysis showed that high TPα expression is associated with a significantly shorter BCR-free time in cases with organ-confined pT2 staging (Supplementary Figure S5B; *P* = 0.0007, *n* = 105), but not in extra-prostatic/invasive pT3 cases (Supplementary Figure S5B; *P* = 0.8798, *n* = 104). In contrast, for TPβ, multivariate analysis showed that high TPβ expression is associated with a significantly shorter BCR-free time in cases with invasive pT3 staging (Supplementary Figure S5C; *P* = 0.0293, *n* = 107), but not in organ-confined pT2 cases (Supplementary Figure S5C; *P* = 0.8229, *n* = 109). Hence, these additional multivariate analyses suggest that expression of TPα and of TPβ may indeed provide independent prognostic value over and above that provided solely through histological Gleason scoring or pathological tumour staging.

### Analysis of the methylation profile of the promoter regions of the TBXA2R gene in benign prostate and PCa tissue

**Figure 4:**
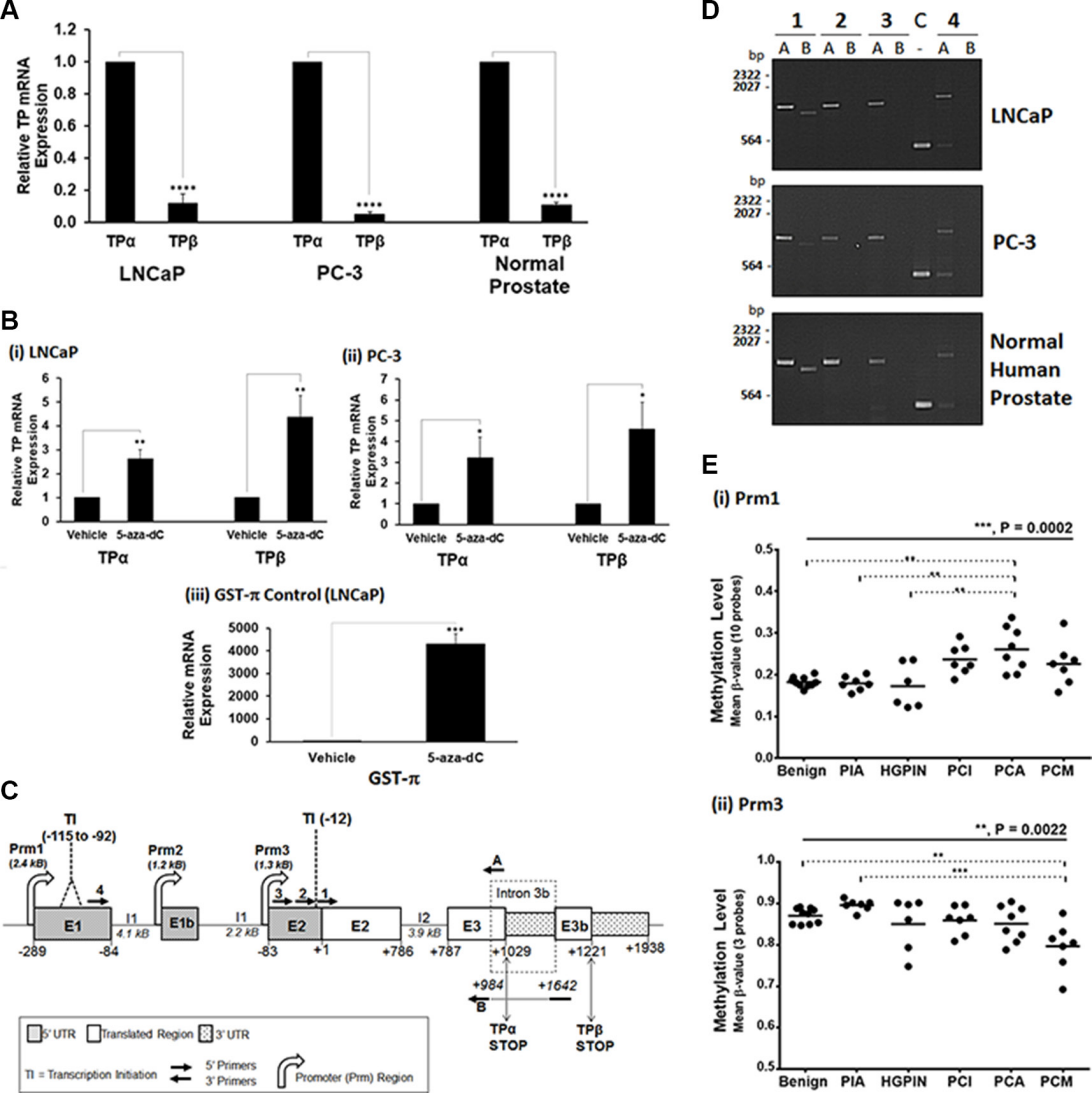
Transcriptional regulation & methylation of the TBXA2R in PCa Panel (**A**) qRT-PCR analysis of TPα and TPβ mRNA expression levels in LNCaP, PC-3 cells and normal human prostate. All data was normalized relative to 18S rRNA levels. Data are presented as mean relative TPα or TPβ mRNA expression (± SEM, *n* ≥ 3). *Panel* (**B**) qRT-PCR analysis of TPα and TPβ mRNA expression levels in LNCaP (i) and PC-3 (ii) cells, along with GST-π mRNA expression levels in LNCaP (iii) cells, treated with 5 μM 5-Aza-dC at 24-hr intervals for 5 days or, as control, with vehicle (0.005 % DMSO). Data were normalized relative to 18S rRNA levels in each case, and are presented as mean expression (± SEM, *n* ≥ 3) relative to those levels found in the vehicle-treated cells. *Panel* (**C**) A schematic of the human *TBXA2R* gene showing the relative positions of exon (E)1, E1b, E2, E3, E3b, intron (I) 1, I2, I3b and the 3 promoters (Prm)1, Prm2 and Prm3, located 5′ of E1, E1b and E2, respectively. The position of the major transcription initiation (TI) sites on the TPα (−115 to −92, within EI) and TPβ (−12, within E2) mRNAs, and of the 5′ and 3′ untranslated regions (UTR) in addition to the translated regions within the TPα and TPβ mRNAs are also shown. All nucleotide numbering is assigned relative to the translation start site ATG at nucleotide +1. *Panel* (**D**): Agarose gel electrophoresis of RT-PCR products derived from total RNA extracted from PC-3 and LNCaP cells or normal human prostate tissue. To determine which promoter (Prm1 or Prm3) is driving TPα and/or TPβ mRNA expression, a series of 5′ primers located within E1 (Primer 4) or E2 (Primers 1, 2 or 3) were used in PCR experiments in combination with the TPα (Primer A) or TPβ (Primer B) specific 3′ primers. For TPα, amplicons were generated for all 5′ primers (Primers 1−4) used in combination with Primer A (TPα specific), but in the case of TPβ, amplicons were generated with the 5′ primer Primer 1, but not Primers 2-4, in combination with Primer B (TPβ specific) showing that the major transcription initiation (TI) sites of TPα mRNA mapped to the TI within EI (~ −115 to – 92) but for the TPβ mRNA mapped to the TI (at ~ −12) within E2. The control (C) lane shows amplicons for GAPDH. *Panel* (**E**): Data from hybridization analysis of the Infinium Human Methylation 450K Beadchip with CpG probe sets for the (i) Prm1 or (ii) Prm3 regions of the TXBA2R gene. Scatter plots show the distribution of methylation levels (β values) in LCM-dissected specimens from benign tissues (*N* = 10), proliferative inflammatory atrophy (PIA; *N* = 7) and high-grade prostatic intraepithelial neoplasia (HGPIN; *N* = 6) specimens, and from indolent (PCI; *N* = 7), aggressive (PCA; *N* = 8) and metastatic (PCM; *N* = 7) PCa samples. Differences were analysed by one-way ANOVA group comparisons (solid black line) and Tukey's multiple comparisons (dotted lines). In all panels, *, **, *** and **** indicate *P* < 0.05, 0.01, 0.001 and 0.0001, respectively.

CpG genomic DNA methylation through either hypermethylation or hypomethylation, particularly within the regulatory promoter elements, acts to regulate gene expression and plays a major role in cancer development through transcriptional silencing or activation of critical tumour suppressor genes or oncogenes, respectively [38]. Bearing in mind findings herein from two independent TMAs that expression of TPα and TPβ isoforms are significantly but differentially altered as a function of both PCa staging and progression (time to disease-free/BCR-free survival), we sought to investigate whether altered CpG methylation of the *TBXA2R* gene might play a role in the transcriptional regulation of TPα and TPβ, potentially accounting for the observed differences in TPα and TPβ expression in PCa. Hence, a RT-PCR based approach was initially used to quantify relative TPα and TPβ mRNA expression in human prostate tissue or in the prostate adenocarcinoma LNCaP and PC-3 cell lines and to determine whether their expression can be regulated in response to genomic demethylation. Quantitative RT-PCR confirmed expression of both TPα and TPβ mRNA within the prostate-derived RNA and that there was, on-average, a 10-fold higher level of TPα than TPβ mRNA expressed (Figure 4A). Furthermore, global genomic DNA demethylation with 5-Aza-2′-deoxycytidine (5-Aza-dC) led to significant increases in both TPα (2.8 -fold) and TPβ (4.5-fold) mRNA expression, and of the control GST-π mRNA-amplicon, in the adenocarcinoma LNCaP and PC-3 cell lines (Figure 4B), confirming that the *TBXA2R* gene may be regulated by CpG methylation.

As stated while TPα and TPβ are encoded by the same *TBXA2R* gene, they are transcriptionally regulated by two distinct promoters [26, 27] whereby Prm1, located 5′ of the transcription initiation (TI; nucleotide −289) site in exon/E1, exclusively regulates TPα while Prm3, located 5′ of the TI (nucleotide −12) within exon/E2, exclusively regulates TPβ expression (Figure 4C). Hence, a RT-PCR based approach was also used to clarify which promoters within the *TBXA2R*, namely Prm1 or Prm3, are regulating TPα and TPβ expression in human prostate tissue or in the prostate adenocarcinoma LNCaP and PC-3 cell lines. Consistent with previous findings in megakaryocytes [26, 27], Prm1 and Prm3 were confirmed to exclusively regulate TPα and TPβ expression, respectively, in the human prostate tissue and adenocarcinoma cell lines as evidenced by the finding that the 5′-most nucleotide (TI site) of TPα mRNA mapped to exon/E1 while the 5′-most nucleotide of TPβ mRNA mapped to its TI site at nucleotide −12 located within exon/E2 (Figure 4C and 4D).

Thereafter, the CpG methylation profile of the Prm1 and Prm3 regions of the *TBXA2R* was assessed by interrogating the Infinium HumanMethylation450 BeadChip for changes in DNA methylation as a function of PCa progression (Figure 4E). Specifically, methylation levels of genomic DNA extracted from prostate tissue, dissected by laser-capture microscopy (LCM), from a subset of 45 PCa patients mainly selected from the PCRC (Dublin-based) TMA ranging from benign/BPH (*N* = 10) through to the progressive stages of PCa including proliferative inflammatory atrophy (PIA; *N* = 7), high-grade prostatic intraepithelial neoplasia (HGPIN; *N* = 6), indolent (PCI; *N* = 7), aggressive (PCA; *N* = 8) and metastatic (PCM; *N* = 7) PCa samples was evaluated by hybridization (Prm1, with 10 specific CpG probes; Prm3, with 3 specific CpG probes). While Prm1 (regulates TPα mRNA expression) was found to lie within a hypomethylated region in the benign/BPH and PIA lesions (β value ≈ 0.2), with little variation between individuals, methylation of Prm1 increased significantly in pre-invasive HGPIN tissues and primary and metastatic lesions (*P* = 0.0002, ANOVA). Whilst the increased Prm1 methylation is highly significant in the aggressive cohort (*P* < 0.01; Figure 4E(i)), overall methylation intensities remain low (β < 0.5) within this Prm1 region of the *TBXA2R* (Figure 4E(i)). In dramatic contrast to Prm1, the Prm3 region which regulates TPβ expression is highly hypermethylated in benign and precursor PIA lesions (β value ≈ 0.8), and there were significant decreases in Prm3 methylation observed within the metastatic cohort relative to the benign and precursor (PIA) lesions (*P* = 0.0022, ANOVA; Figure 4E(ii)). Collectively, these data show highly significant differences in the CpG methylation status of Prm1 and Prm3 of the *TBXA2R* in both the benign/BPH tissue and also during the transition and progression of PCa, whereby the changes that occur in PCa begin at the HGPIN stage and result in a *nett* increased methylation of Prm1 and a *nett* decreased methylation of Prm3, potentially accounting for the variable expression of TPα in PCa and the more global increase in TPβ expression as a function of Gleason grade.

## DISCUSSION

Prostate carcinogenesis and tumour development involves several critical steps including cell proliferation, survival, migration, invasion, and ultimately metastasis. Recently, several lines of evidence point to the role of the TXA_2_-TP signalling axis in cancer progression, not least from longitudinal studies showing that long-term daily use of aspirin reduces the risk of many common cancers (predominantly gastrointestinal, but also breast, lung, and prostate cancers), with numerous clinical trials completed/underway testing its benefits and of other NSAIDs/COXIBs in chemoprevention [6, 39, 40]. Increased levels of TXA_2_ and expression of its synthase (TXA_2_ synthase) and its receptor, the TP, have been implicated in a number of prevalent cancers [8], including in bladder [41], prostate [12, 13], colorectal [14, 15] and non-small cell lung cancer [16]. However, while clinical evidence for the role of the TXA_2_-TP axis in cancer progression is increasing, with the exception of bladder cancer [11] previous studies have not investigated the role of the individual TPα or TPβ isoforms in cancer or indeed in other diseases in which TXA_2_ has been implicated. While TPα and TPβ are encoded by the same gene, they are differentially expressed being under the control of different promoters [24–27] within the *TBXA2R*, and appear to be functionally distinct displaying both common/over-lapping but also isoform-specific physiologic roles. Although showing similar coupling to Gq/phospholipase Cβ and to Gq/G12-RhoA [10, 18], TPα and TPβ differentially regulate other effectors including the PRKs [18, 19] and are themselves differentially regulated [22, 42–44]. Critically and in this regard, signalling by TPα, but not TPβ, is completely inhibited by the counter-regulatory *anti-*platelet and vasodilatory agents prostacyclin and nitric oxide mediated by direct protein kinase (PK) A and PKG phosphorylation of TPα at Ser^329^ and Ser^331^, respectively, the very first divergent residues between TPα and TPβ [43, 44]. The conclusion from those studies is that TPα is essential for haemostasis/thrombosis, while the role of TPβ in this pathophysiologic process remains less clear [43, 44].

Mechanistically, the ability of the TXA_2_-TP axis to regulate tumour development and metastasis can be explained by the ability of TPα/TPβ to regulate key mitogenic/ERK- and RhoA-mediated signalling cascades [9, 10] and, potentially, by its ability to regulate local inflammation and immunity [45–50]. Furthermore, and in the context of PCa, TPα and TPβ differentially regulate signalling by the PRKs, a RhoA effector known to act as a specific *gate-keeper* of androgen receptor (AR) -mediated chromatin remodelling (H3pThr11) and hence, androgen-dependent gene expression within the prostate [19, 32–34]. In fact, due to the ability of the PRKs to regulate androgen-induced gene expression even in the absence of androgens, such as in situations that may mimic androgen deprivation therapy (ADT) in the clinical setting, aberrant activation of the PRKs has been proposed as one possible cellular mechanism that leads to CRPC, the metastatic lethal form of PCa that occurs on failure of *anti-*androgen therapies [4]. Hence, the discovery that the TXA_2_-TP signalling axis can directly activate the PRKs to induce the essential chromatin modifications (H3pThr11) necessary to support AR-mediated gene expression within the prostate even in the absence of androgens *per se* led us to interrogate the role of TXA_2_ in PCa, including in CRPC [19, 35].

In the current study, we evaluated the expression profiles of both TPα and TPβ in TMAs representative of benign prostate tissue and of the different Gleason grades (3, 4, and 5) of PCa obtained following prostatectomy through IHC staining using isoform-specific antibodies. The majority of *anti-*TPα immunoreactivity within the prostate epithelium was cytoplasmic. In addition, abundant TPα expression was also evident within the smooth muscle cells of the fibromuscular stromal regions, consistent with the recognised contractile role of TXA_2_ in the prostate [51]. While less abundant than TPα within the prostate stroma, the majority of *anti-*TPβ immunoreactivity within the luminal epithelium was cytoplasmic, but substantial nuclear staining of TPβ was also evident in several specimens. Predominant cytoplasmic expression/IHC staining for TPα and TPβ is not unexpected bearing in mind that both TPs undergo agonist-induced internalization [44, 52] while under tonic/resting conditions, a substantial component of both TPα and TPβ localise to the endoplasmic reticulum and Golgi complex [53]. Furthermore, at any given time, a proportion of TPβ is stored in intracellular pools of perinuclear recycling endosomes, from which they may be subsequently trafficked to the cell surface as required [54]. Noteworthy, whilst nuclear expression of TPβ > TPα was also evident in certain cores of the TMAs used in the current study, there was no correlation between nuclear expression of either TP with any of the pathological parameters (Gleason grade or BCR) examined.

Initially, through the use of the “PCRC TMA” and borne out by findings from the “Malmö TMA” and from a third independent Dublin-based TMA of 61 PCa patients across 450 tissue-cores (data not shown), cytoplasmic expression of the TPβ isoform was found to be significantly increased in prostate tumours relative to benign tissue, with increasing expression of TPβ significantly correlating with an increase in Gleason grade and pathological stage. Furthermore, while expression of TPα was found to be variably increased or decreased across all 3 TMAs screened within this study, there was no overall direct correlation between TPα expression with Gleason grade or pathological stage observed. These findings involving 3 independent TMAs, totalling 529 PCa cases and 2841 benign/malignant cores, are entirely in keeping with the independent expression/transcriptional regulation of TPα and TPβ [24–27]. Notably, a previous study conducted on full-face PCa tissue samples from a small patient cohort (*N* = 46) using an antibody which does not discriminate between the individual TPα/TPβ isoforms found that expression of the TP was increased in the malignant relative to benign tissue, with advanced pathological stages and poorly-differentiated tumours having the highest expression levels [12].

While the vast majority of prostate tumours remain asymptomatic [1, 55], following surgery or radiation therapy approximately one third of patients with localised PCa experience disease relapse, in what is diagnostically termed “biochemical recurrence (BCR)” or PSA failure [3]. Hence, using the “Malmö TMA” for which 10-yr follow-up clinical records are available, the association between TPα and TPβ expression with patient-progression to BCR was investigated. Strikingly, high levels of TPβ expression were associated with significantly increased risk of BCR and shorter BCR-free survival time following surgery. For TPα, while not quite achieving statistical significance using the binary low/high scoring approach, there was a considerable trend with ‘high TPα expression’ levels being associated with BCR (*P* = 0.057) and shorter BCR-free survival time (*P* = 0.0776). However, the alternative ordinal scoring approach showed that expression of TPα does in fact correlate with time-to-progression to BCR (*P* = 0.0291). The fact that the correlation between high TPα expression and BCR or BCR-free survival time did not reach significance using all approaches is not surprising given that the relative ratio of low/decreased *versus* high/increased expression of TPα in the tumour regions was evenly distributed (50:50). However, it was notable that those patient cases with high TPα expression also showed a strong correlation with high TPβ expression (*P* = 0.0001; Table 2C) showing that increased expression of either TPα, TPβ or both TP isoforms trends toward the poorer outcome. In line with that suggestion, while 34% (*P* = 0.0396) of patients in the Malmö TMA displaying high levels of TPβ in the tumour progressed to BCR, that figure increased to 41% (*P* = 0.0017) in patients showing both high TPα and TPβ expression in the tumour region, where in both instances the mean time to progression to BCR was 42–43 months post-surgery. Taken together, these findings suggest that increased expression of TPβ, or of TPα or a combination of TPα/TPβ expression levels, may serve as diagnostic biomarkers and clinical predictors of PCa disease recurrence. Noteworthy, additional multivariate analyses herein suggested that expression of TPα and of TPβ may indeed provide independent prognostic value over and above that provided solely through histological Gleason scoring or pathological tumour staging. Confirming the predictive power of these findings is indeed advisable through investigation in other independent patient cohorts, including with additional or longer term follow-up clinical histories or in TMAs that might enable combined correlation of TP expression with Gleason grade, tumour staging and BCR. However, based on the findings from this study, it is indeed possible to suggest that employing a similar approach (i.e. using TPα and/or TPβ isoform specific antibodies), expression of TPα and/or TPβ could be histologically assessed at an early stage post-diagnosis or post-radical prostatectomy to improve the diagnostic and prognostic evaluation of patients with PCa and can help stratify patients with a higher likelihood of progressing to disease relapse/BCR, who would require additional therapies. At the same time, in conjunction with the use of other histological biomarkers, the application of this strategy may help to avoid unnecessary therapies, and the associated complications thereof, in patients deemed to be at a lower risk of PCa recurrence. While our histologic evaluations were based on TMAs derived from radical prostatectomies, additional evaluations of biopsy samples from PCa patients may enable clarification on whether assessment of TPα and/or TPβ expression might also be used to as a useful biomarker for determining whether a patient may require radical prostatectomy or not.

In normal mammalian development, CpG DNA methylation/demethylation serves to modulate gene expression [56], and plays a major role in cancer through the transcriptional silencing or activation of critical growth regulators such as tumour suppressor genes or oncogenes, respectively [38]. As stated, expression of TPα is under the control of Prm1 within the *TBXA2R* gene, while TPβ is under the control of Prm3 [26, 27]. While a recent study revealed that the tumour suppressors Wilms' tumour (WT) 1 and hypermethylated in cancer (HIC)1 transcriptionally regulate TPα expression through binding to *cis*-elements within Prm1 in both prostate and breast cancer cell lines [57], to the best of our knowledge no study has yet investigated the mechanisms governing the transcriptional regulation of TPβ through Prm3 in similar settings. Herein, it was confirmed that Prm1 and Prm3 exclusively regulate TPα and TPβ mRNA expression, respectively, both in normal human prostate and in the PCa adenocarcinoma LNCaP and PC-3 cell lines and that expression of both mRNAs' can be increased in response to global genomic demethylation with 5-Aza-dC. Bearing in mind our findings that the histological expression of TPα and TPβ are significantly but differentially altered as a function of PCa staging and progression, but that increased expression of either isoform is specifically associated with disease relapse (BCR) and shorter BCR-free survival time, it was of interest to investigate the patterns of DNA methylation within similar PCa cohorts. Significant changes in the methylation landscape within the Prm1 and Prm3 regions of the *TBXA2R* were observed across a range of benign, precursor prostate neoplasms and increasing PCa staging. Specifically, and consistent with the higher level of TPα mRNA expression in the prostate and clonal cell lines, it was found that Prm1 is hypomethylated in benign tissues and precursor lesions but that it undergoes increased methylation across primary and metastatic PCa stages potentially accounting for the overall reduced level of TPα such as generally observed across the “Malmö TMA”. Conversely, the Prm3 region is highly hypermethylated in benign and precursor lesions, with significant losses of methylation observed within certain aggressive and metastatic PCa samples, potentially accounting for the overall increased expression of TPβ as a function of Gleason grade and associated with both TMAs. These investigations clearly show that the *TBXA2R* gene is subject to altered CpG methylation as a function of PCa staging. Moreover, they prompt detailed methylation sequencing analysis of the *TBXA2R* to determine whether the alterations in TPα and/or TPβ expression observed in PCa tissue is due to altered methylation of specific *cis*-acting elements within Prm1 or Prm3 controlling TPα and TPβ transcriptional expression. It is indeed notable that increased expression of TPβ, without concomitant changes in TPα expression have been found in clinically derived tissue from patients with high-grade bladder cancer (*N* = 43) and high levels of TPβ in those patients correlated with reduced overall survival [11].

In conclusion, data presented herein propose a novel diagnostic and prognostic potential for TPα and TPβ in the histopathological assessment of PCa. While TPα and TPβ are differentially expressed and independently regulated, increased levels of TPβ, TPα or both correlated with increased risk of recurrence and reduced disease-free survival time. Moreover, correlation of TPα and TPβ expression with pathological staging showed what high TPα expression was significantly associated with the organ-confined pT2 cases while, in stark contrast, high TPβ expression was significantly associated with the invasive pT3 cases. We also show that altered CpG methylation of the Prm1 and Prm3 regions of *TBXA2R* gene can specifically modulate TPα and TPβ expression, respectively, during the progression of PCa. Following detailed investigation of the *trans*-acting transcription factors regulating Prm1/TPα and Prm3/TPβ in PCa settings, interrogation of the CpG methylation status of the *TBXA2R* might also serve as a key clinical marker with which to stratify higher risk patients and to tailor treatment options. Taken together, these studies provide significant credence to the functional role of the TXA_2_-TP signalling axis in cancer progression and to the growing body of evidence for the prophylactic benefits of aspirin in reducing cancer risk by lowering TXA_2_ levels. Whether there is a particular added clinical benefit of aspirin therapy in those patients showing elevated TPβ or TPα needs to be considered. Furthermore, investigation of the therapeutic potential of targeting TPβ and/or TPα through the use of receptor antagonists, such as in adjuvant therapy, for PCa treatment may also be warranted.

## MATERIALS AND METHODS

### Tissue specimens and tissue microarrays

Full-face formalin-fixed and paraffin-embedded radical prostatectomy samples (*N* = 17) were obtained through ethical consent from St. Vincent's University Hospital (Dublin, Ireland). Tissue microarrays (TMAs) from two distinct patient cohorts were used. The first TMA (“PCRC TMA”, Table 3A), used to investigate TPα and TPβ immunohistochemical (IHC) staining/expression with Gleason grading, was constructed from a population-based cohort of 130 PCa patients who underwent radical prostatectomy at one of three Dublin (Ireland) hospitals and collected as part of the Prostate Cancer Research Consortium (PCRC) bioresource. At the point of diagnosis, these specimens were reviewed and graded according to the Gleason scoring system. Cores from these cases included a variety of histological Gleason scores, ranging from 6 to 10, where three cores of each histological stage (Benign/BPH and Gleason grade 3, 4 and 5 carcinomas) were taken from each case, where available. The second TMA (“Malmö TMA”, Table 3B), used to investigate correlation of TPα and TPβ expression with disease recurrence, was constructed from radical prostatectomy samples from 338 PCa patients who underwent surgery at Skåne University Hospital (Malmö, Sweden), and for which clinical follow-up data (minimum 10 year follow-up) and pathological staging is also available. Cores taken from this patient cohort included a variety of histological Gleason scores, ranging from 5 to 10, where two tissue cores were taken from each of the benign, primary (predominant) and secondary Gleason grade patterns, where available. The use of tissue samples was approved by the respective Ethics Committee of the participating institutions.

**Table 3A:** “PCRC TMA” – characteristics and composition

Number of Patients	130
Patient Age (Mean ± SD)	61.2 ± 6.53 yrs
PSA At Surgery (Mean ± SD)	8.35 ± 3.49 ng/ml
**Gleason Scoring**	**Number^$^**
6	34
7 (3 + 4)	39
7 (4 + 3)	25
8	21
9	11
**Clinical Staging**	**Number^$^**
T2a/b	11
T2c	57
T3a	47
T3b	15
**TMA Composition**	**Number^#^**
Total Cores	1038
Mean Cores/Patient	7.9
Benign/BPH Cores	389
Gleason 3 Cores	320
Gleason 4 Cores	272
Gleason 5 Cores	45
Miscellaneous/Other^†^	12

Footnotes:

$ refers to the number of patient cases.

# refers to the number of cores.

† refers to patient cases that could not be graded and thus eliminated from all statistical analyses.

**Table 3B:** “Malmö TMA” - characteristics and composition

Number of Patients	338
Patient Age (Mean ± SD)	62.9 ± 5.75 yrs
PSA At Surgery (Mean ± SD)	9.21 ± 6.05 ng/ml
# Patients with Recurrence (%)	99 (30%)
Time to Recurrence (Mean ± SD)	44.61 ± 30.86 months
**Gleason Scoring**	**Number^$^**
5	34
6	110
7 (3 + 4)	107
7 (4 + 3)	54
8	4
9	20
10	1
Not Known^†^	8
**Pathological Staging**	**Number^$^**
pTX	14
pT2	165
pT3	157
pT4	2
**TMA Composition**	**Number^#^**
Total Cores	1355
Mean Cores/Patient	4.1

Footnotes:

$ refers to the number of patient cases.

# refers to the number of cores.

† refers to patient cases that could not be graded and thus eliminated from all statistical analyses.

### Immunohistochemistry

Serial sections of formalin fixed, paraffin embedded full-face prostate tissue or TMA blocks were sectioned at 4 μm thickness and baked onto slides at 50–56°C for at least 60 min. Sections were dewaxed in two changes of xylene (2 × 10 min) and rehydrated through a series of decreasing alcohol solutions (100%, 3 min × 2; 95%, 1 min; 80%, 1 min) before being washed in distilled water. Endogenous peroxidase activity was blocked by incubation in 3% hydrogen peroxide, prepared in methanol, for 10 min at R.T., followed by washing of tissue sections in PBS. Non-specific binding was blocked by incubating the tissue sections for 30 min at R.T. with 5% goat serum in PBS (Blocking Buffer) to which Avidin D (4 drops/ml of Blocking Buffer; Vector Labs Avidin/Biotin Blocking kit) was added to block endogenous biotin. Thereafter, sections were incubated overnight at 4°C in a humidified chamber with primary (1°) antibody, prepared in Blocking Buffer and containing Biotin (4 drops/ml of Blocking Buffer; Vector Labs Avidin/Biotin Blocking kit), where affinity-purified rabbit *anti-*TPα (12 μg/ml) or *anti-*TPβ (1.3 μg/ml) antibodies [10, 36] were used to detect TPα or TPβ. As controls, either the 1° antibody was omitted, or the 1° *anti-*TPα or *anti-*TPβ antibodies were pre-incubated at R.T. for 1 h with a 5-fold (w/w) excess of either the immunogenic TPα (aa 329–343) or TPβ (aa 391–407) peptides or a randomised non-competitor peptide. Sections were washed in PBS, prior to incubation for 30 min with biotinylated goat *anti-*rabbit secondary antibody (1:500 dilution), prepared in Blocking Buffer. After washing, tissue sections were incubated with streptavidin horseradish peroxidase (HRP; 1 in 1500, prepared in Blocking buffer) for 30 min at room temperature, followed by incubation with the chromogen 3,3′-diaminobenzidine (DAB) substrate (0.05% DAB, 0.015% hydrogen peroxide in PBS) for 1–5 min. Tissue sections were counterstained with haematoxylin, dehydrated through increasing alcohol series and xylene (2 × 10 min) prior to mounting in DPX. Slides were imaged using a Zeiss microscope and AxioVision software or digitally scanned using the Aperio ImageScope system.

### TMA scoring & statistical analysis

Each TMA core was reviewed to verify the presence of benign tissue or adenocarcinoma and to annotate the Gleason grade of the tumour, where applicable (C.S./E.W.K.). Cytoplasmic immunoreactivity/expression of TPα and TPβ in the benign and tumour prostate epithelium across both TMAs used in the study was independently assessed by two pathologists (C.S./E.W.K.). For the purpose of statistical analysis, expression in 10% or more cells was required for a positive score, and was graded according to the following scale: 0, no staining; 1, faint but clearly detectable staining in > 10% of epithelial cells; 2, moderate staining in > 10% of epithelial cells; and 3, strong staining in > 10% of epithelial cells. Occasionally, in cases where there was marked variability, an intermediate staining intensity score (*i.e.* 0.5, 1.5, 2.5) was assigned. Some unusable cores were found in the TMAs due to missing tissue, cancer necrosis, or insufficient cancer cells and, hence, these cores were excluded from the study. The staining intensities of TPα and TPβ in the cytoplasm of the benign and tumour epithelium was then further divided into two groups: “low expression” (score < 2) included those with negative or weak staining and “high expression” (score ≥ 2) included those with moderate or strong reactivity. Statistical analyses of differences were carried out using one-way ANOVA followed by post hoc Dunnett's multiple comparison t tests, as indicated. Fisher exact tests were performed on 2 × 2 contingency tables to test association of TPα/TPβ IHC score (high expression [≥ 2] and low expression [< 2]) with Gleason grading. Kaplan–Meier analyses were used for survival comparisons to test association of TPα/TPβ IHC score (high expression [≥ 2] and low expression [< 2]) with progression to BCR. All values are expressed as mean ± standard error of the mean (SEM). *P*-values < 0.05 were considered to indicate statistically significant differences, where *, ** and *** indicate *P* < 0.05, *P* < 0.01 and *P* < 0.001, respectively. Statistical analyses were performed using GraphPad Prism, version 5.00.

### Reverse transcriptase (RT)-PCR & real-time quantitative RT-PCR (qRT-PCR) analysis

The human PCa adenocarcinoma PC-3 and LNCaP cell lines, obtained from the American Type Culture Collection and confirmed mycoplasma-free, were routinely cultured at 37°C in a humid atmosphere with 5% CO_2_ as described previously [19]. Human prostate total RNA (ThermoFisher Scientific) or total RNA, extracted from PCa cell lines using TRIzol reagent (Sigma), was DNase-treated and was converted to first-strand (1°) cDNA with SuperScript^®^ III Reverse Transcriptase (Invitrogen), as previously described [26, 27]. For TPα/TPβ-typing studies, conventional non-quantitative/end-point reverse transcriptase (RT)-PCR analysis was performed essentially as previously described [26, 27], where the specific primers and respective target regions are detailed in Supplementary Table S1A.

To quantify TPα or TPβ mRNA expression or investigate the effect of alterations in DNA CpG methylation on TPα or TPβ mRNA expression in response to global genomic DNA demethylation, PC-3 or LNCaP cells were incubated for 5 days with either 5 μM 5-Aza-2′-deoxycytidine (5-Aza-dC; Sigma) or, as controls, with vehicle (0.005 % DMSO), where in all cases drug/vehicle was replaced every 24 hr. Cells were then harvested, total RNA extracted (TRIzol reagent) and converted to 1° cDNA as previous. Real-time quantitative RT-PCR (qRT-PCR) analysis was performed using the SyBr Green reaction kit, as previously described [57], where the primers and respective target regions are detailed in Supplementary Table S1B. As a positive control, changes in glutathione *S*-transferase (GST)-π mRNA expression levels in LNCaP cells were quantified using TaqMan technology using a gene-specific expression assay (Hs00168310.m1; Applied Biosystems). To obtain ΔCt values, levels of TPα, TPβ and GSTP1 mRNA expression were normalized relative to 18S rRNA levels. ΔΔCt values were calculated using control/vehicle ΔCt values. Relative TPα, TPβ and GST-π mRNA expression levels were calculated using the formula 2^−ΔΔCt^ [58], and data is presented as mean changes in mRNA expression relative to control levels, assigned a value of 1 (Relative expression ± SEM, *n* ≥ 3). Results were obtained from RNA extracted from three independent PC-3 or LNCaP cell treatments, with multiple independent cDNAs generated from each treatment, and where qRT-PCR samples were run in duplicate in all cases.

### Genomic methylation analysis

Tissues used to investigate the CpG methylation status of the *TBXA2R* gene as a function of PCa progression were ethically sourced via the Prostate Cancer Research Consortium (PCRC) from one of three Dublin (Ireland) hospitals or, for the metastatic patient cohort, from the University of Washington Medical Center via their Prostate Cancer Donor Program. Six pathological stages were chosen to reflect the progression of PCa; benign prostate (*N* = 10), proliferative inflammatory atrophy (PIA; *N* = 7), high-grade prostatic intraepithelial neoplasia (HGPIN; *N* = 6), indolent (PCI; *N* = 7), aggressive (PCA; *N* = 8) and metastatic (PCM; *N* = 7) PCa stages. Benign tissues were obtained from FFPE radical cystoprostatectomy specimens with no evidence of PCa, while PIA, HGPIN and PCa samples were obtained from FFPE radical prostatectomy specimens. Indolent tumours were defined as Gleason grade 3, pT2, pre-operative PSA ≤ 10 ng/ml with no evidence of biochemical or clinical recurrence. Aggressive tumours were defined as Gleason grades 4/5, pT3, with seminal vesicle and/or lymph node involvement and biochemical recurrence. Metastatic tumours were sampled from multiple tissue sources (liver, lymph node and adrenal). Laser capture microdissection was performed using the Arcturus XT to enrich for prostatic epithelial cells in each sample. Genomic DNA was extracted using the QIAamp DNA micro kit and was quantified using Qubit fluorometric analysis. DNA isolated from the microdissected samples underwent FFPE damage restoration (Infinium) and bisulphite modification (Zymo Research) prior to processing on the Infinium HumanMethylation450 BeadChip Assay platform (Illumina, Inc., San Diego, CA) according to manufacturer's instructions. Methylation call data were subsequently analyzed using the RnBeads pipeline [59]. Differences were analysed by one-way ANOVA group comparisons and Tukey's multiple comparisons.

## SUPPLEMENTARY MATERIALS TABLES FIGURES



## Abbreviations

5-Aza-dC: 5-Aza-2′-deoxycytidine
ADT: androgen deprivation therapy
AR: androgen receptor
BCR: biochemical recurrence
BPH: benign prostatic hyperplasia
COX: cyclooxygenase/prostaglandin G2/H2 synthase
CRPC: castrate resistant prostate cancer
ERK: extracellular signal regulated protein kinase
FBS: foetal bovine serum
GS: Gleason scoring
H3Thr11: histone H3 threonine 11
HEK: human embryonic kidney
HGPIN: high-grade prostatic intraepithelial neoplasia
IHC: immunohistochemistry
NSAID: non-steroidal anti-inflammatory drug
PCa: prostate cancer
PIA: prostatic inflammatory atrophy
PK: protein kinase
PRK: protein kinase C-related kinase
Prm: promoter
PSA: prostate-specific antigen
PTS: pathological tumour staging
TMA: tumour microarray
TP: T prostanoid receptor
TXA2: thromboxane (TX) A2
WT1: Wilms' tumour (WT) 1
