# Supplementary material for: Expression of the TPα and TPβ isoforms of the thromboxane prostanoid receptor (TP) in prostate cancer: clinical significance and diagnostic potential
Source: Oncotarget. 2016 Sep 26;7(45):73171–87. doi: 10.18632/oncotarget.12256 (PMC5341971; doi:10.18632/oncotarget.12256)
Supplement: Supplementary file 1 [file oncotarget-07-73171-s001.pdf]

Expression of the TPα and TPβ isoforms of the thromboxane prostanoid receptor (TP) in prostate cancer: clinical significance and diagnostic potential

Supplementary Materials

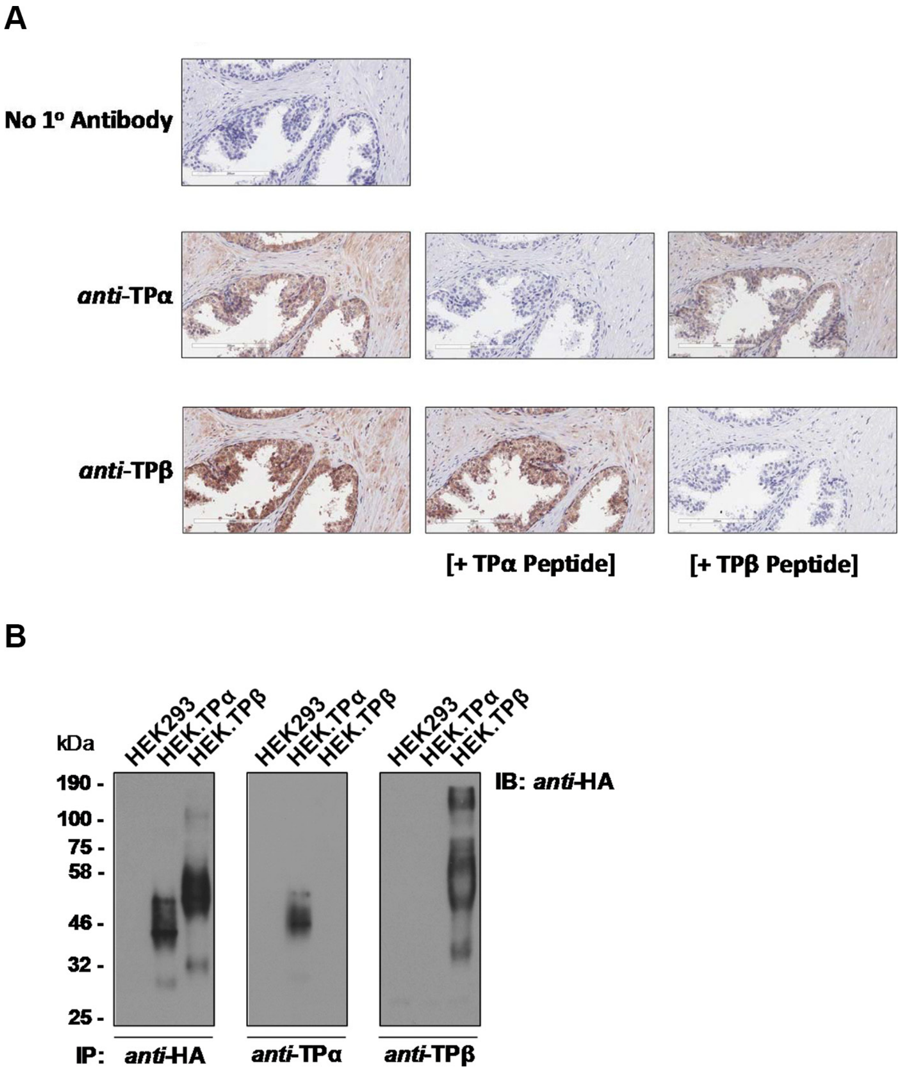

C

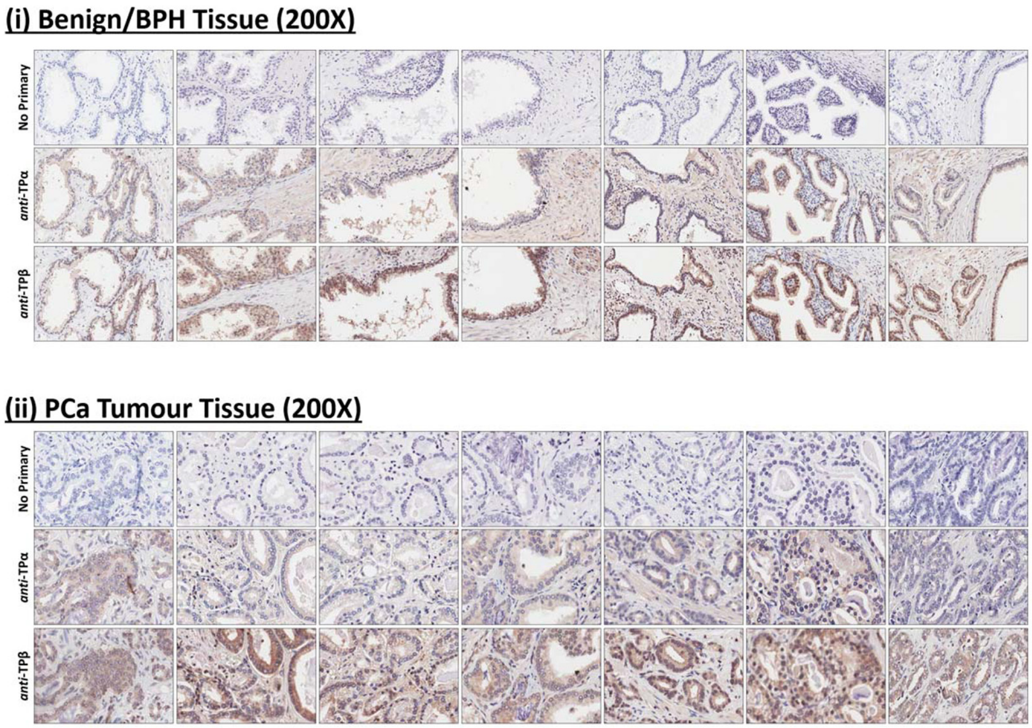

Supplementary Figure S1: Validation of TPα and TPβ antibodies. *Panel* (**A**) IHC analysis of serial sections of paraffin-embedded, formalin-fixed prostate tissue screened either in the absence of primary antibody or with affinity-purified *anti*-TPα or *anti*-TPβ antibodies. The specificity of the *anti*-TPα antibody was confirmed whereby the cognate antigenic TPα peptide, but not a TPβ peptide, abolished *anti*-TPα specific staining. Similarly, the specificity of the *anti*-TPβ antibody was confirmed whereby the antigenic TPβ peptide, but not the TPα peptide, abolished *anti*-TPβ specific staining. All sections were counterstained with haematoxylin and images shown were captured at 200× magnification. *Panel* (**B**) Lysates from HEK 293, HEK.TPα or HEK.TPβ cells expressing haemagglutinin (HA)-tagged TPα or TPβ, respectively, were each subject to immunoprecipitation using *anti*-HA (101R) or with affinity-purified *anti*-TPα or *anti*-TPβ antibodies (4 µg sera per immunoprecipitation). Immunoprecipitates (IP) were then resolved by SDS-PAGE and immunoblotted (IB) with *anti*-HA (3F10-POD) antibody, as indicated. The relative positions of the molecular size markers (kDa) are indicated. *Panel* (**C**) Representative IHC analysis of TPα and TPβ protein expression in serial sections of paraffin-embedded, formalin-fixed full-face prostate tissues screened with affinity-purified *anti*-TPα or *anti*-TPβ antibodies. In all, 17 full-face sections were examined where representative images of the (i) benign and (ii) tumour regions from 7 of those specimens is shown. All sections were counterstained with haematoxylin and images shown were captured at 200× magnification.

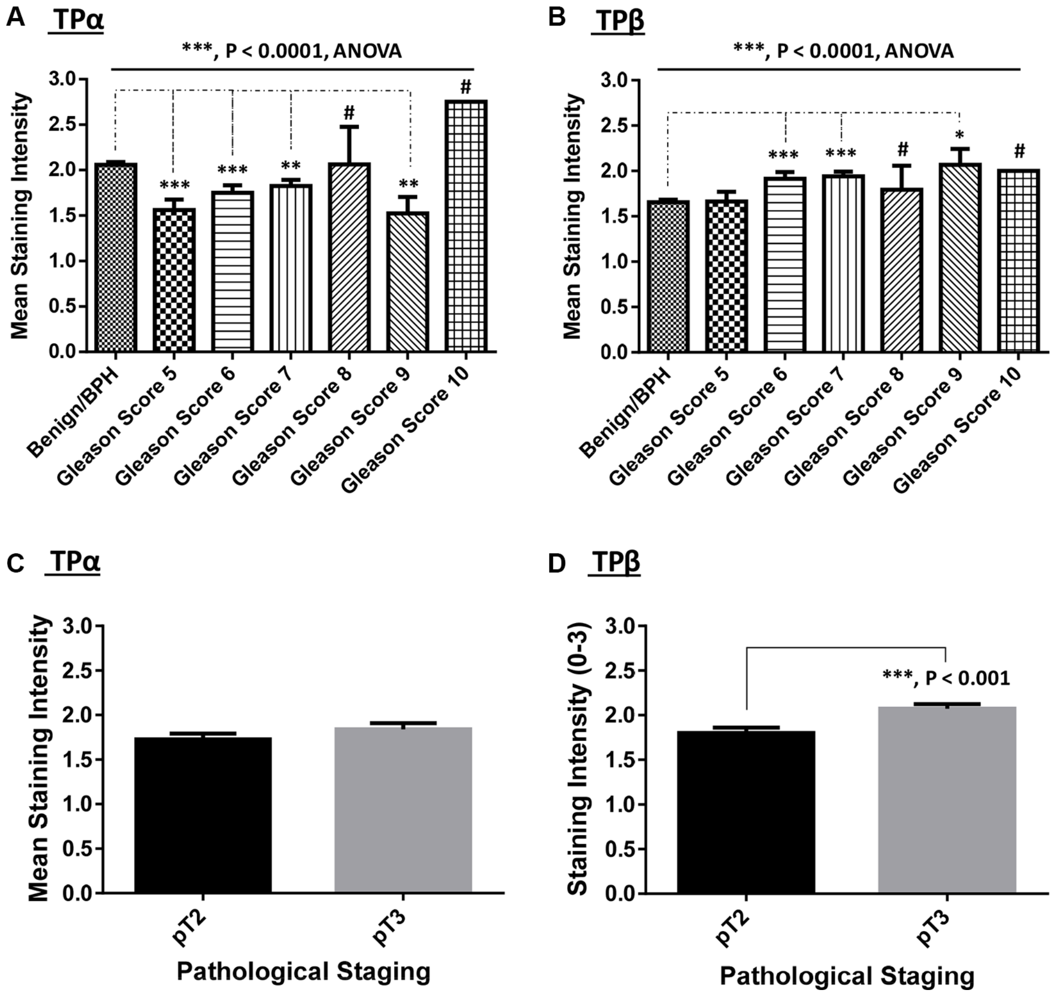

Supplementary Figure S2: Correlation of TPα and TPβ staining intensity with gleason scoring and pathological staging in the malmo TMA. *Panels A & B:* Comparison of TPα (*Panel* **A**) and TPβ (*Panel* **B**) immunohistochemical staining intensities in the "Malmo TMA", where data is presented as the mean TPα or TPβ score (Range, 0-3) for each particular histology (Gleason Score) present (± SEM). Differences were analysed by one-way ANOVA group comparisons (solid black line) with *post-hoc* Bonferroni's Multiple Comparison Tests (dotted line) and the asterisks indicate where scoring of TPα (*Panel A*) or TPβ (*Panel B*) staining intensity within the PCa tissue is significantly altered between the groups indicated, or compared to the Benign/BPH tissue, where *, ** and *** indicate $P < 0.05$, 0.01 and 0.001, respectively. The symbol # indicates where *n* numbers for the particular Gleason score groupings were below levels required for valid statistical evaluations; specifically, for Gleason score 8 ($n = 4$) and for Gleason score 10 ($n = 1$), respectively. *Panels C & D:* Comparison of TPα (*Panel* **C**) and TPβ (*Panel* **D**) immunohistochemical staining intensities in the "Malmo TMA", where data is presented as the mean TPα or TPβ score (Range, 0-3) for each particular Pathological Stage present (± SEM). Differences were analysed by Student's t-test and the asterisk indicates where scoring of TPβ (*Panel D*) staining intensity within the PCa tissue is significantly altered compared to the Benign/BPH tissue, where *** indicates $P < 0.001$.

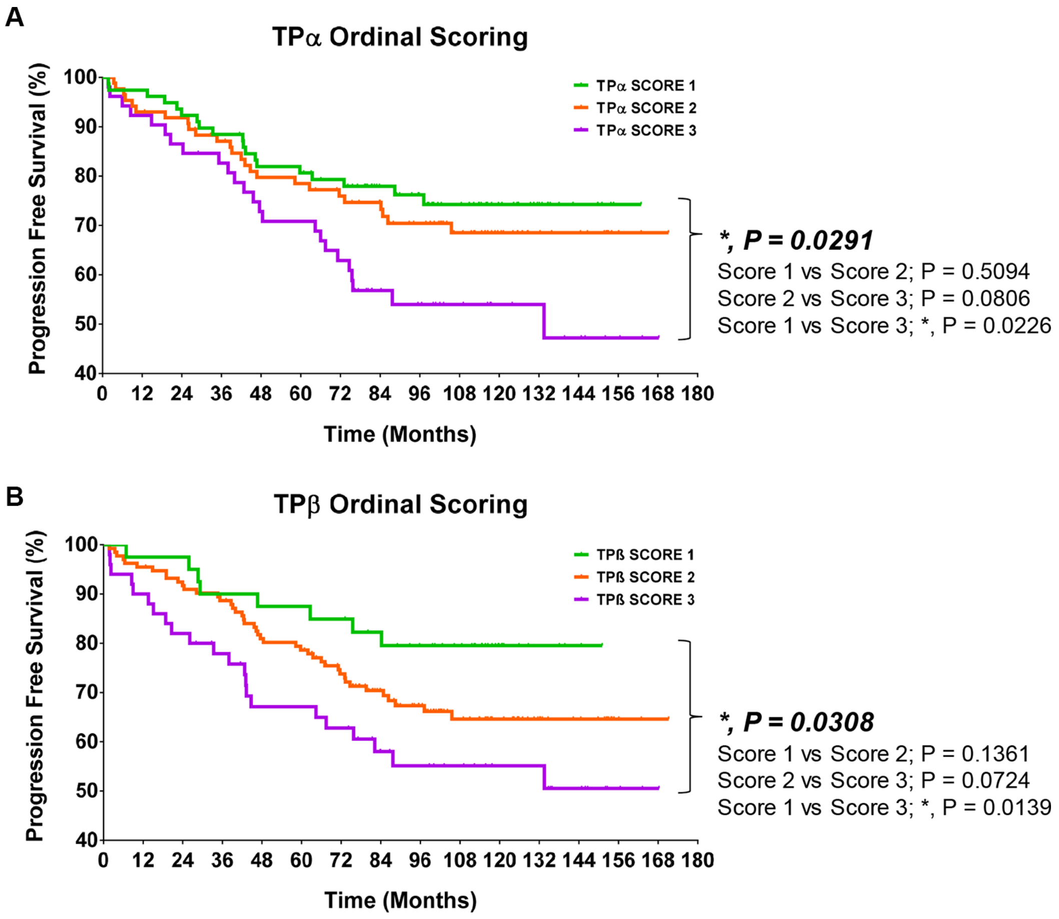

Supplementary Figure S3: Correlation of TPα and TPβ expression with the progression to BCR. Kaplan–Meier survival analysis of the correlation between ordinal scoring values of TPα (*Panel* **A**) and TPβ (*Panel* **B**) expression with time to onset of BCR using the "Malmö TMA" dataset. BCR-free survival was compared between the expression groups by Kaplan–Mantel–Cox log rank comparison with Gehan–Breslow–Wilcoxon correction test between the individual groupings, where * indicates $P < 0.05$.

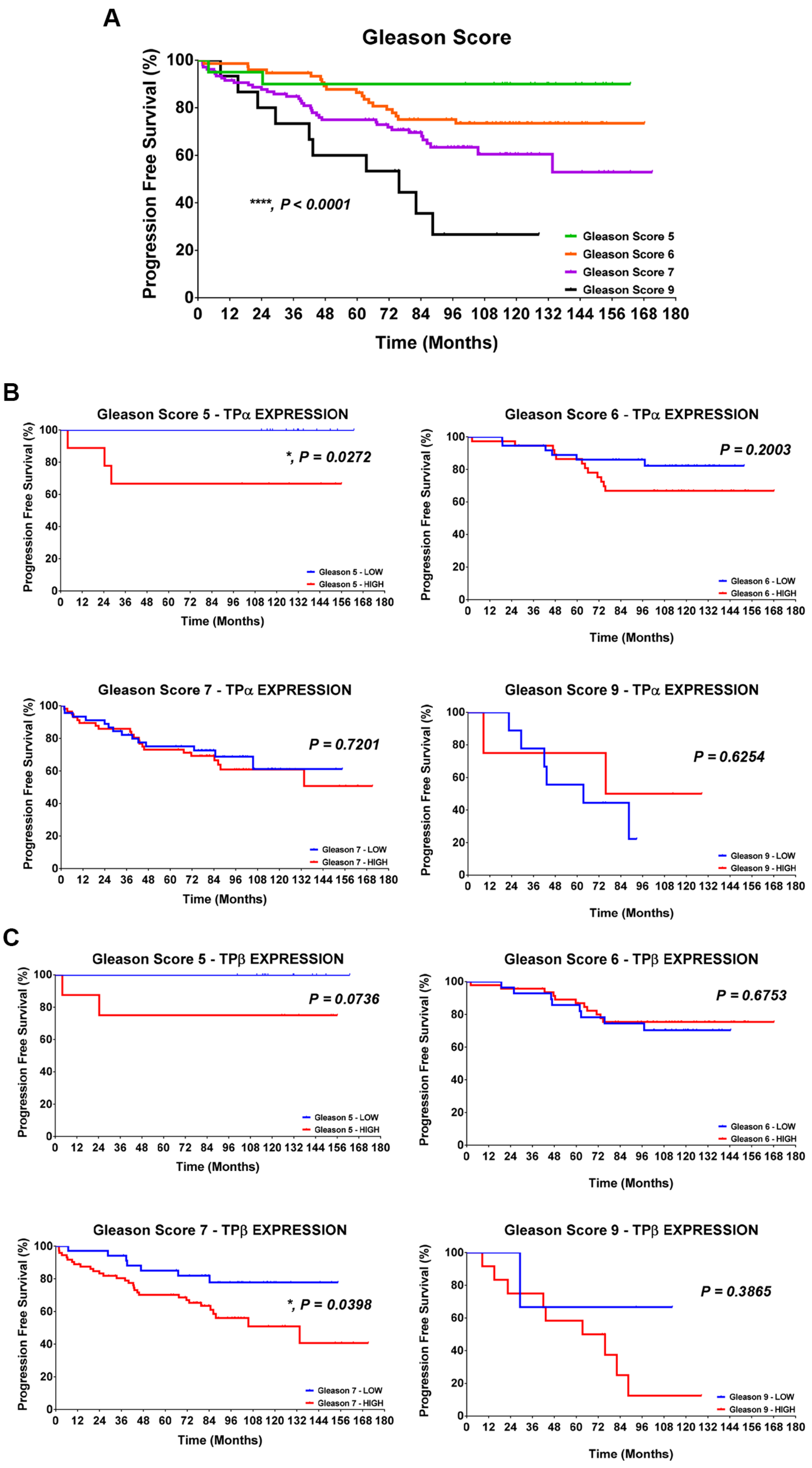

Supplementary Figure S4: Multivariate correlation of gleason scoring and TPα/TPβ expression with the progression to BCR. Panel (**A**) Kaplan–Meier survival analysis of the correlation between Gleason scoring with time to onset of BCR using the "Malmö TMA" dataset. Panels B & C: Kaplan–Meier survival analysis of the correlation between low or high levels of TPα (*Panel* (**B**) and TPβ (*Panel* (**C**) expression, in groupings of cases with the indicated Gleason score, with time to onset of BCR using the "Malmö TMA" dataset. BCR-free survival was compared between the expression groups by Kaplan–Mantel–Cox log rank comparison with Gehan–Breslow–Wilcoxon correction test between the individual groupings, where * indicates $P < 0.05$.

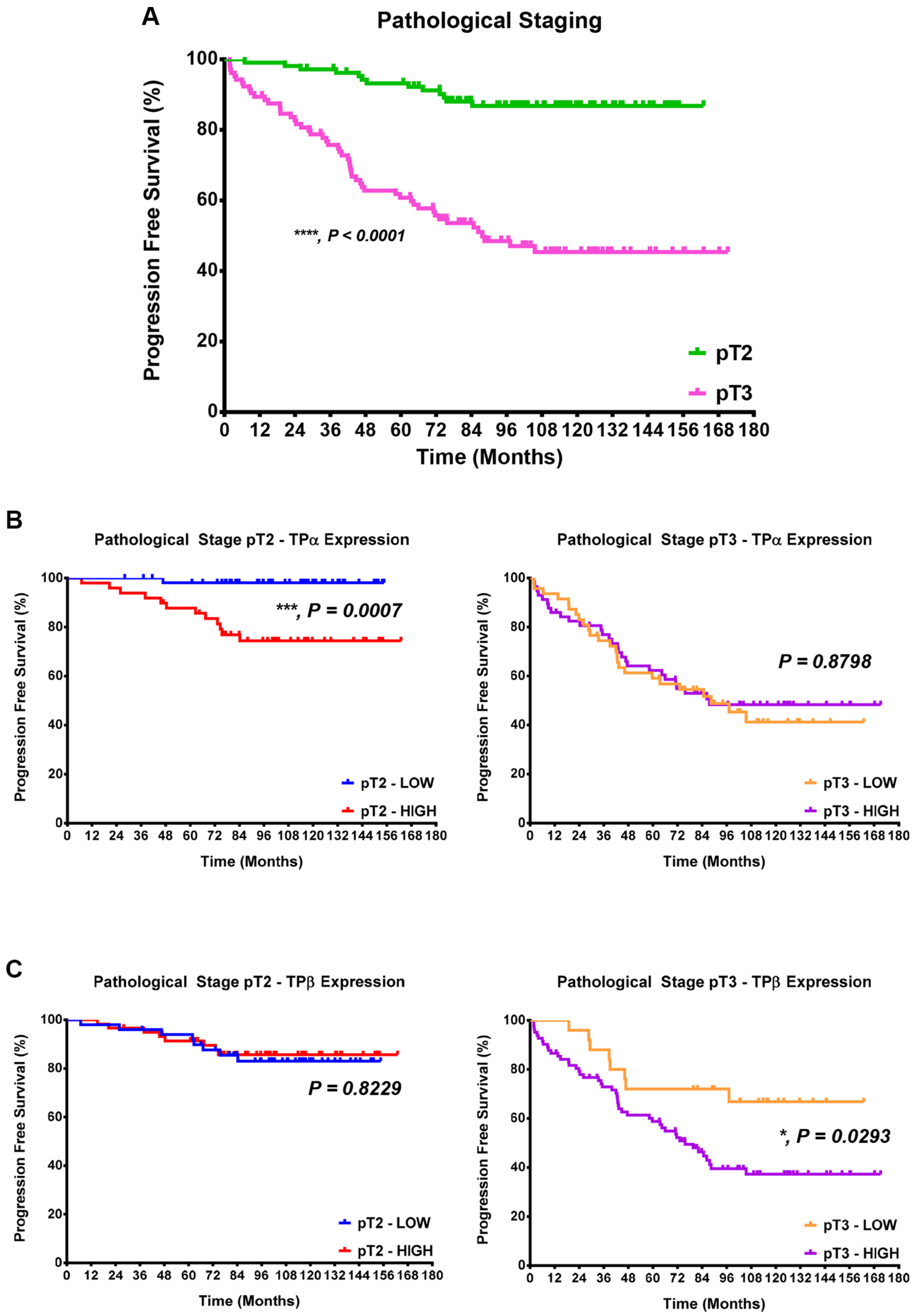

Supplementary Figure S5: Multivariate correlation of pathological staging and TPα/TPβ expression with the progression to BCR. Panel (**A**) Kaplan–Meier survival analysis of the correlation between pathological staging with time to onset of BCR using the "Malmö TMA" dataset. Panels (**B & C**) Kaplan–Meier survival analysis of the correlation between low or high levels of TPα (*Panel B*) and TPβ (*Panel C*) expression, in groupings of cases with the indicated pathological staging, with time to onset of BCR using the "Malmö TMA" dataset. BCR-free survival was compared between the expression groups by Kaplan–Mantel–Cox log rank comparison with Gehan–Breslow–Wilcoxon correction test between the individual groupings, where * indicates $P < 0.05$.

Supplementary Table S1A: "Malmo TMA" - TPα staining intensity/gleason scoring correlation

Contingency Test	Histology	Low Expression No. (%)	High Expression No. (%)	P Value$
**Histologically benign versus tumour (Gleason score 5–10)**	Benign/BPH ($n^{\#}$ = 325)	88 (27)	237 (73)	***$P < 0.0001$
	Gleason score 5–10 (n = 224)	113 (50)	111 (50)	
**Histologically benign versus Gleason score 6–10**	Benign/BPH (n = 325)	88 (27)	237 (73)	***$P < 0.0001$
	Gleason score 5–10 (n = 201)	100 (50)	101 (50)	
**Histologically benign versus Gleason score 7–10**	Benign/BPH (n = 325)	88 (27)	237 (73)	***$P < 0.0001$
	Gleason score 7–10 (n = 124)	61 (48)	64 (52)	

Supplemental Table S1B: "Malmo TMA" - TPβ staining intensity/gleason scoring correlation

Contingency Test	Histology	Low Expression No. (%)	High Expression No. (%)	*P* Value[$]
Histologically benign versus tumour (Gleason score 5–10)	Benign/BPH ($n^{\#}$ = 323)	182 (56)	141 (44)	***$P < 0.0001$
	Gleason score 5–10 (n = 231)	86 (37)	145 (63)	
Histologically benign versus Gleason score 6–10	Benign/BPH (n = 323)	182 (56)	141 (44)	***$P < 0.0001$
	Gleason score 5–10 (n = 209)	73 (35)	136 (65)	
Histologically benign versus Gleason score 7–10	Benign/BPH (n = 323)	182 (56)	141 (44)	***$P < 0.0001$
	Gleason score 7–10 (n = 131)	43 (32)	89 (68)	

Supplemental Table S1C: "Malmö TMA" - TP staining intensity/pathological stage correlation

Contingency Test	Pathological Stage	Low Expression No. (%)	High Expression No. (%)	*P* Value[$]
**TPα: Stage pT2 vs pT3**	Stage pT2 ($n^{\#}$ = 105)	56 (46)	49 (47)	$P = 0.2694$
	Stage pT3 (n = 104)	47 (45)	57 (55)	
**TPβ: Stage pT2 vs pT3**	Stage pT2 (n = 109)	50 (46)	59 (54)	***$P = 0.0006$
	Stage pT3 (n = 107)	25 (23)	82 (77)	

Footnotes:
[#]*n* refers to the number of valid graded scores in the benign and tumour regions for patient cases.
[$]Fisher's Exact Test where *** refers to $P < 0.001$.

Supplementary Table S2A: Primers used in RT-PCR

Amplicon Name	Template	Forward (5′) Primer #1–4	Reverse (3′) Primer: A (TPα) or B (TPβ)	Amplicon Size (bp)
1-A	TPα mRNA	#1: 5′-GGCTCCGGAGCCATGTG-3′	**#A:** 5′-CCAGCCCCTGAATCCTCA-3′	1133
1-B	TPβ mRNA		**#B:** 5′-AGACTCCGTCTGGGCCG-3′	1003
2-A	TPα mRNA	**#2:** 5′-TGACTGATCCCTCAGGG-3′	**#A:** 5′-CCAGCCCCTGAATCCTCA-3′	1148
2-B	TPβ mRNA		**#B:** 5′-AGACTCCGTCTGGGCCG-3′	1019
3-A	TPα mRNA	**#3:** 5′-CCTGATGGGGTGGTGAC-3′	**#A:** 5′-CCAGCCCCTGAATCCTCA-3′	1161
3-B	TPβ mRNA		**#B:** 5′-AGACTCCGTCTGGGCCG-3′	1032
4-A	TPα mRNA	**#4:** 5′-GCCCTCGCCCCACCCTCG-3′	**#A:** 5′-CCAGCCCCTGAATCCTCA-3′	1319
4-B	TPβ mRNA		**#B:** 5′-AGACTCCGTCTGGGCCG-3′	1190
C	GAPDH mRNA	5′-TGAAGGTCGGAGTCAACG-3′	5′-CATGTGGGCCATGAGGTC-3′	467

Supplementary Table S2B: Primers used in qRT-PCR

Amplicon Name	Template	Forward (5′) Primer*	Reverse (3′) Primer: I (TPα) or II (TPβ)	Amplicon Size (bp)
TPα	TPα mRNA	5′-GCCAGCGTGTGTTGGCT GC-3′	**#I:** 5′-CTGGGGCTGGCGGGACAG-3′	297
TPβ	TPβ mRNA		**#II:** 5′-GAGACTCCGTCTGGGCCG-3′	234
18S rRNA	18S rRNA	5′-AACCCGTTGAACCCATT-3′	5′-CCATCCAATCGGTAGTAGCG-3′	149

Footnotes:

*, Common TPα/TPβ forward primer, corresponding to nucleotides 759–778 of Exon 2 within the TP gene.

#I, TPα-specific reverse primer corresponding to nucleotides 1006–1988 of TPα mRNA.

#II, TPβ-specific reverse primer corresponding to nucleotides 992–974 of TPβ mRNA.

SUPPLEMENTARY MATERIALS AND METHODS

Immunoprecipitations

Human embryonic kidney (HEK) 293 and HEK.TPα, HEK.TPβ cells, stably overexpressing haemagglutinin (HA)-tagged forms of TPα and TPβ, respectively, have been previously described [1]. Prior to immunoprecipitation, cells were washed twice in ice-cold PBS and incubated in Radioimmune Precipitation (RIP) buffer (20 mM Tris-Cl, pH 8.0, 150 mM NaCl, 10 mM EDTA, 1% (v/v) NP-40, 1% (w/v) sodium deoxycholate, 0.1% (w/v) SDS, 1 mM sodium orthovanadate, 1 mM PMSF, 4 µg/ml leupeptin, 2.5 µg/ml aprotinin; 800 µl/10 cm dish) on ice for 10 min to lyse the cells. Lysates were homogenised by passing through needles of decreasing gauge (21–26) and clarified by centrifugation at 13,000 *g* for 5 min to pellet the cell debris. The resulting supernatants were subject to immunoprecipitation, using *anti*-HA 101R (4 µg; Covance) or affinity purified *anti*-TPα (4 µg) and *anti*-TPβ (4 µg) specific antibodies, to pull-down the relevant protein through overnight incubation at 4°C on a rotator. Thereafter, the lysates were incubated for 1 hr with Protein G-Sepharose (*anti*-HA immunoprecipitations; 50% slurry in RIP buffer; 15 µl) or Protein A-Sepharose (anti-TPα/TPβ immunoprecipitations; 50% slurry in RIP buffer; 30 µl), prior to washing with at least four changes of RIP buffer followed by four changes of PBS. Immunoprecipitates were then subjected to immunoblotting versus anti-HA 3F10-HRP antibody.

REFERENCES

1. Turner EC, Kavanagh DJ, Mulvaney EP, McLean C, Wikstrom K, Reid HM, Kinsella BT. Identification of an interaction between the TPalpha and TPbeta isoforms of the human thromboxane A2 receptor with protein kinase C-related kinase (PRK) 1: implications for prostate cancer. J Biol Chem. 2011; 286:15440–15457.
